# Highly efficient emerging Ag_2_BaTiSe_4_ solar cells using a new class of alkaline earth metal-based chalcogenide buffers alternative to CdS

**DOI:** 10.1038/s41598-024-51711-6

**Published:** 2024-01-17

**Authors:** Kaviya Tracy Arockiya Dass, M. Khalid Hossain, Latha Marasamy

**Affiliations:** 1 Facultad de Química, Materiales-Energía, Universidad Autónoma de Querétaro, 76010 Santiago de Querétaro, QRO México; 2 Institute of Electronics, Atomic Energy Research Establishment, Bangladesh Atomic Energy Commission, Dhaka, 1349 Bangladesh

**Keywords:** Solar cells, Electronic devices

## Abstract

Cu_2_ZnSn(S,Se)_4_ is a non-toxic, earth-abundant photovoltaic absorber. However, its efficiency is limited by a large open circuit voltage (V_OC_) deficit occurring due to its antisite defects and improper band alignment with toxic CdS buffer. Therefore, finding an absorber and non-toxic buffers that reduce V_OC_ deficit is crucial. Herein, for the first time, Ag_2_BaTiSe_4_ is proposed as an alternative absorber using SCAPS-1D wherein a new class of alkaline earth metal chalcogenide such as MgS, CaS, SrS, and BaS is applied as buffers, and their characteristics are compared with CdS to identify their potential and suitability. The buffer and absorber properties are elucidated by tuning their thickness, carrier concentration, and defect density. Interestingly, optimization of the buffer’s carrier concentration suppressed the barrier height and accumulation of charge carriers at the absorber/buffer interface, leading to efficiencies of 18.81%, 17.17%, 20.6%, 20.85%, 20.08% in MgS, CaS, SrS, BaS, and CdS-based solar cells respectively. Upon optimizing Ag_2_BaTiSe_4,_ MoSe_2_, and interface defects maximum efficiency of > 28% is achieved with less V_OC_ loss (~ 0.3 V) in all solar cells at absorber’s thickness, carrier concentration, and defect density of 1 µm, 10^18^ cm^−3^, 10^15^ cm^−3^ respectively, underscoring the promising nature of Ag_2_BaTiSe_4_ absorber and new alkaline earth metal chalcogenide buffers in photovoltaics.

## Introduction

The world’s population is growing at a rapid pace, and with technological advancements, there is a higher demand for energy in all sectors. Fossil fuels have been the primary source of energy, but their excessive usage is leading to environmental problems due to toxic gas emissions, and they are also becoming scarce^1,2^. This has caused a need for renewable sources of energy to cater to the increasing demand. Scientists have been researching the generation of solar energy through photovoltaic devices to produce electricity^3^. The most widely used solar cells are made from crystalline silicon, which has a high conversion efficiency and is thermally stable, besides being abundant in the earth’s crust. However, their manufacturing cost is high because of the energy required for production^4^. Other materials used for solar cells such as CdTe and CIGSe have a lower payback period, are less expensive to produce, and have a higher efficiency, but their commercialization is limited due to their toxicity (Cd) or scarcity (In and Ga)^5,6^. CZTSSe of group I_2_-II-IV-VI_4_ is a promising alternative material for solar cells since it is non-toxic, has a high absorption coefficient, and its elements are available in abundance in the earth’s crust^7^. However, CZTSSe solar cells have a lower efficiency of 14.9%, less than their theoretical limit of 31%, resulting from a high V_OC_ deficit^8–10^. This is caused by antisite defects (Cu_Zn_ and Zn_Cu_), arising from the similar ionic size of Cu and Zn^11,12^. Through various experiments, researchers have attempted to suppress the antisite defects and enhance the efficiency of CZTSSe solar cells by altering its elements (Cu/Zn). For instance, Cu_2_CdSnS_4_ material demonstrated the highest PCE of over 10% where the toxicity of Cd is the major problem^13^. Other materials such as Cu_2_FeSnS_4_, Cu_2_BaSn(S,Se)_4_, Cu_2_SrSnS_4_, Cu_2_CoSnS_4_, Cu_2_NiSnS_4_, Cu_2_MnSnS_4_, Ag_2_ZnSnS_4_, and n-Ag_2_ZnSnSe_4_ were also studied^14–21^. But, the overall efficiency of these materials still falls short of CZTSSe solar cells.

Currently, scientists are exploring ways to enhance the efficiency of I_2_-II-IV-VI_4_ materials that are used in solar cells. Interestingly, they have discovered that a large density of Sn_Cu_ and Sn_Zn_ antisite defects are also present in CZTSSe solar cells, which can have an adverse impact on their performance by increasing the recombination of charge carriers^22^. These defects are believed to be responsible for the V_OC_ deficit in these solar cells, indicating the need to modify group IV (Sn) elements in the cells along with group I and II (Cu/Zn) to enhance their efficiency. To address this issue, researchers have suggested Ag_2_BaTiSe_4_ as an alternative material^23^. This non-toxic and earth-abundant material possesses a suitable bandgap of 1.18 eV and remarkable optoelectronic properties. It exhibits a strong optical response in the visible and NIR regions, and the probability of the formation of group I, II, and IV elements-based antisite defects is comparatively low^23^. However, despite its ideal absorber properties, its applicability in photovoltaics has not been explored theoretically or experimentally. Therefore, further research is necessary to investigate its material characteristics and photovoltaic performance.

Furthermore, improper band alignment between absorber and buffer drastically affects the V_OC_ of I_2_-II-IV-VI_4_ solar cells^9^. CdS is a typical buffer that is used in many solar cells, but it contains toxic Cd, which makes it difficult to use in an industrial setting^24^. Researchers have tried using alternatives such as ZnMgO, ZnS, ZnO, Zn(S,O), and In_2_S_3_ where the native point defects, large series resistance, high interface recombination, difficulty in controlling the S-to-O ratio along with secondary phase formation, and the presence of rare earth element (In) are found to be their critical problems respectively^25–29^. There are non-toxic and earth-abundant options such as MgS, CaS, SrS, and BaS, which are alkaline earth metal-based chalcogenide semiconductors^30^. These materials have low reflectance, low absorbance, and high transmittance in the visible region, making them ideal buffers for thin-film solar cells. Moreover, they can be easily synthesized^31–35^. Although they have promising properties, it is not yet clear whether they are suitable for use as buffers in thin-film solar cells. This presents an excellent opportunity for the photovoltaic community to explore the properties of these materials as potential buffers in solar cells.

Scientists have used theoretical simulations to improve the design of solar cells by studying how different materials affect their performance^36^. The software used for this purpose is called Solar Cell Capacitance Simulator in One Dimension (SCAPS-1D), which can model solar cells and provide crucial information about how each layer of the cell influences its performance. This information includes details about the materials’ properties, band alignment, interfacial defects, resistance, and the solar cell's stability^37^. SCAPS-1D has been used to simulate various types of solar cells, and its results have been consistent with experimental outcomes^37–39^.

In our study, we used SCAPS-1D to explore the properties and functions of solar cells made with a new absorber called Ag_2_BaTiSe_4_ and new alkaline earth metal chalcogenide buffers such as MgS, CaS, SrS, and BaS. We also designed solar cells with the conventional CdS buffer to compare the performance of different buffers. We analyzed the impact of various factors on the solar cells, such as the thickness, carrier concentration, defect density of buffers, and Ag_2_BaTiSe_4_ absorber layer. We also investigated the role of Ag_2_BaTiSe_4_’s electron affinity and the influence of interfacial defects, parasitic resistance and working temperature on the solar cells' performance. Our research provides valuable insights into the potential of alkaline earth metal chalcogenides as alternative buffers and the properties of Ag_2_BaTiSe_4_ for the development of non-toxic, low-cost, and efficient thin-film solar cells.

## Device structure and method

SCAPS-1D (version 3.3.10) is a solar cell simulation software used to extensively study the properties of each layer in the solar cell^40^. It was developed by Marc Burgelman in the Department of Electronics & Information Systems at the University of Gent, Belgium^41^. It is widely utilized to simulate various solar cells, including CdTe, CIGSe, CZTS, perovskites, etc.^42–44^. It assists the photovoltaic community by providing current density–voltage (J–V), capacitance–voltage (C–V), capacitance–frequency (C–F), quantum efficiency (QE), energy band diagram, carrier density, electric field, and recombination profiles of the modeled solar cells. In addition, other output parameters such as V_OC_, short circuit current density (J_SC_), fill factor (FF), and PCE corresponding to each layer’s parameters can be analyzed. It performs these operations by solving three fundamental equations: Poisson, continuity, and transport equations of charge carriers integrated in SCAPS-1D software^45^. Poisson (Eq. 1), continuity (Eq. 2 and Eq. 3), and charge transport (Eq. 4 and Eq. 5) equations of charge carriers are provided below:

1 $$\frac{{\partial^{2 } \varphi \left( x \right)}}{{\partial x^{2} }} = \frac{q}{\varepsilon } \left( {n\left( x \right) - p\left( x \right) - N_{D}^{ + } \left( x \right) + N_{A}^{ - } \left( x \right) - p_{t} \left( x \right) + N_{t} \left( x \right)} \right)$$

2 $$\frac{\partial n}{{\partial t}} = \frac{1}{q}\frac{{\partial J_{n} }}{\partial x} + \left( {G_{n} - R_{n} } \right)$$

3 $$\frac{\partial p}{{\partial t}} = - \frac{1}{q}\frac{{\partial J_{p} }}{\partial x} + \left( {G_{p} - R_{p} } \right)$$

4 $$J_{n} = qD_{n} \frac{\partial n}{{\partial x}} - q\mu_{n} n\frac{\partial \varphi }{{\partial x}}$$

5 $$J_{p} = qD_{p} \frac{\partial p}{{\partial x}} - q\mu_{p} p\frac{\partial \varphi }{{\partial x}}$$

where q, ε, p, n, $N_{D}^{ + }$, $N_{A}^{ - }$, G_n_, G_p_, R_n_, R_p_, φ, D_n_, D_p_, J_p_, J_n_, μ_n_, and μ_p_ are elemental charge, dielectric constant, hole concentration, electron concentration, donor-type doping concentration, acceptor-type doping concentration, generation rate of electrons, generation rate of holes, recombination rate of electrons, recombination rate of holes, electric potential, coefficient of electron diffusion, coefficient of hole diffusion, hole current density, electron current density, electron mobility, and hole mobility respectively.

**Figure 1:**
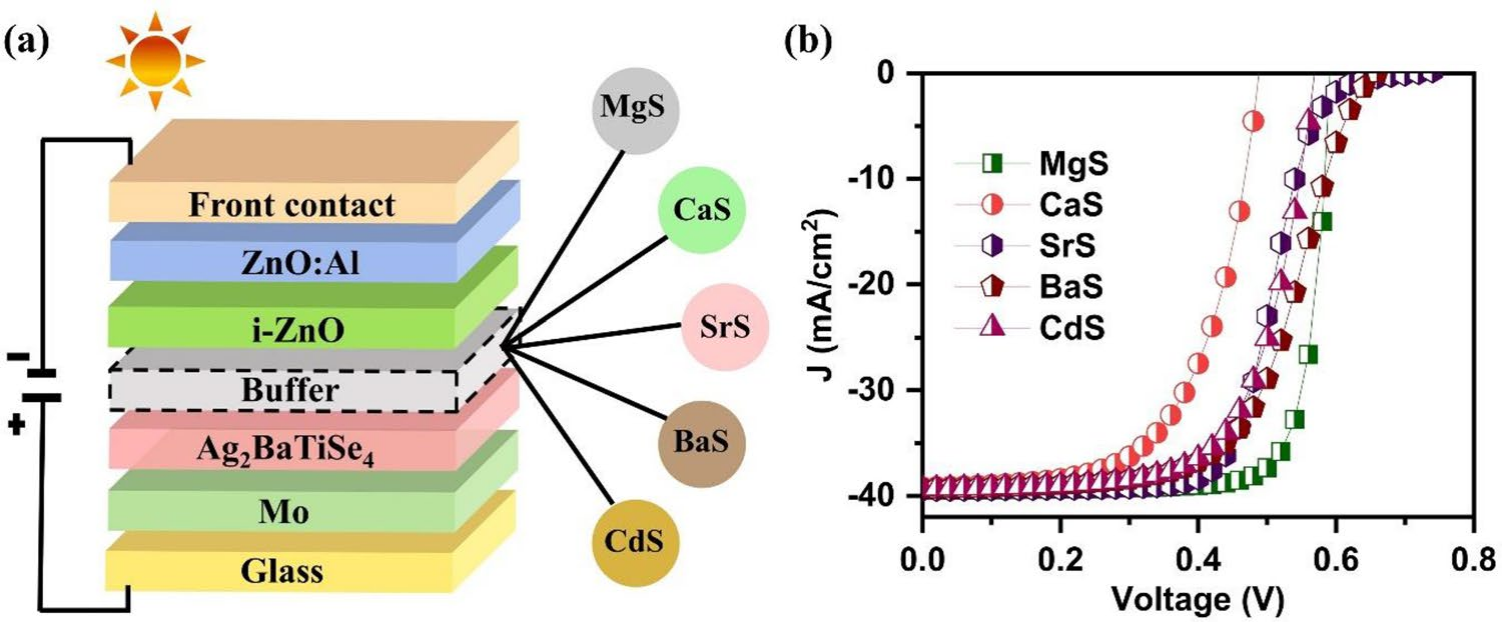
(**a**) Schematic structure of novel Ag_2_BaTiSe_4_ solar cells with diverse buffers. (**b**) Initial J–V of novel Ag_2_BaTiSe_4_ solar cells.

In this work, we have investigated the performance of novel Ag_2_BaTiSe_4_ solar cells with five buffers: MgS, CaS, SrS, BaS, and CdS, using SCAPS-1D. It is simulated in substrate device configuration of front contact/Al:ZnO(AZO)/i:ZnO(IZO)/buffer/Ag_2_BaTiSe_4_/MoSe_2_/Mo/glass as shown in Fig. 1a. The initial parameters of each layer used for the simulation of the solar cell are listed in Table 1. These parameters are taken from the literature^45–50^. In Table 1, E_g,_ χ, ε_r,_ N_C_, N_V_, µ_n_, µ_p,_ N_D_, N_A_, N_t_, SA, and SD represent bandgap, affinity, dielectric permittivity, effective density of states in the conduction band, effective density of states in the valence band, electron mobility, hole mobility, donor concentration, acceptor concentration, defect density, single acceptor, and single donor respectively. The thermal velocity of electrons and holes are fixed at 10^7^ cm s^-1^ for all the layers, and flat band condition is applied to the front contact. The simulations are carried out at 300 K under AM 1.5G spectral irradiance, wherein shunt and series resistance are not considered. In addition, neutral defects are introduced at the Ag_2_BaTiSe_4_/MoSe_2_ and buffer/Ag_2_BaTiSe_4_ interfaces according to the parameters listed in Table 2 to simulate realistic conditions of solar cells.

**Table 1:** Input parameters of different layers of novel Ag_2_BaTiSe_4_ solar cells used in the simulation^45–50^.

Parameters	Al:ZnO	i-ZnO	Ag_2_BaTiSe_4_	MoSe_2_	Buffers				
					MgS	CaS	SrS	BaS	CdS
Thickness (μm)	0.200	0.050	1.0	0.050	0.080	0.080	0.080	0.080	0.080
E_g_ (eV)	3.40	3.40	1.18	1.4	2.7	2.14	2.5	3.0	2.42
χ (eV)	4.60	4.60	4.1	4.12	4.3	4.6	4.0	4.15	4.5
ε_r_	9.00	9.00	6	8.76	10	9	10	15	9
N_C_ (cm^−3^)	2.0 × 10^18^	2.0 × 10^18^	1.0 × 10^19^	2.8 × 10^19^	1.9 × 10^19^	2.9 × 10^19^	1.2 × 10^19^	1.2 × 10^18^	1.8 × 10^19^
N_V_ (cm^−3^)	1.8 × 10^19^	1.8 × 10^19^	1.0 × 10^19^	2.65 × 10^19^	1.0 × 10^18^	1.0 × 10^18^	1.4 × 10^18^	1.4 × 10^19^	2.4 × 10^18^
µ_n_ (cm^2^ Vs^−1^)	150	150	100	100	75	150	100	100	160
µ_h_ (cm^2^ Vs^−1^)	25	25	250	250	50	50	25	25	50
N_A_ (cm^−3^)	0	0	1.0 × 10^15^	1.0 × 10^17^	0	0	0	0	0
N_D_ (cm^−3^)	1.0 × 10^20^	1.0 × 10^17^	0	0	1.0 × 10^17^	1.0 × 10^17^	1.0 × 10^17^	1.0 × 10^16^	1.0 × 10^17^
N_t_ (cm^−3^)	1.0 × 10^15^	1.0 × 10^15^	1.0 × 10^15^	1.0 × 10^15^	1.0 × 10^15^	1.0 × 10^15^	1.0 × 10^15^	1.0 × 10^15^	1.0 × 10^15^
Defect type	SA	SA	SD	SD	SA	SA	SA	SA	SA

**Table 2:** Simulation parameters at the interfaces.

Parameters	Ag_2_BaTiSe_4_/buffer interface	Ag_2_BaTiSe_4_/MoSe_2_ interface
Defect density	1.0 × 10^12^ cm^−3^	1.0 × 10^12^ cm^−3^
Defect type	Neutral	Neutral
Capture cross section for electrons	1E−19 cm^2^	1E−19 cm^2^
Capture cross section for holes	1E−19 cm^2^	1E−19 cm^2^
Energetic distribution	Single	Single
Reference for defect energy level	Above the highest valence band	Above the highest valence band
Energy level with respect to valence band maximum	0.6 eV	0.6 eV

Throughout the simulation, the parameters of AZO and IZO were fixed according to Table 1. The thickness of AZO in thin-film solar cells usually ranges from 0.1 to 0.5 µm^51–56^. However, experimental results have shown that a thickness of 0.2 µm is optimal in terms of low resistivity, high mobility, and carrier concentration^57^. Therefore, we have chosen a thickness of 0.2 µm for the AZO layer. Similarly, for IZO, the optimal thickness for high solar cell performance is between 0.04 and 0.1 µm, as reported by experiments^51,53–55,58^. If the thickness is less than 0.04 µm, it increases the leakage current, while for a thickness greater than 0.1 µm, the series resistance increases and the built-in field reduces, leading to degraded performance^59–61^. Based on material usage and experimental range, we have selected a thickness of 0.05 µm for IZO. Likewise, the other material parameters of AZO and IZO were adapted from the experiments^57,61^. The important parameters of other layers such as buffers, Ag_2_BaTiSe_4_ and MoSe_2_ were tuned to discover their optimum range and understand their influence on the solar cell performance.

To begin with, initial solar cells are designed with the parameters listed in Table 1. After that, the performance of the solar cells is studied as a function of buffer thickness (0.05 to 0.2 µm), carrier concentration (10^12^ to 10^20^ cm^−3^), and defect density (10^12^ to 10^20^ cm^−3^). Further, the material characteristics of novel Ag_2_BaTiSe_4_ are investigated by tuning its electron affinity, thickness, carrier concentration, and defect density from 4.1 to 4.7 eV, 0.1 to 2 µm, 10^12^ to 10^18^ cm^−3^ and 10^12^ to 10^20^ cm^−3^ respectively. Moreover, the role of MoSe_2_’s thickness (0.05–0.2 µm) and carrier concentration (10^12^–10^20^ cm^−3^) are analyzed. After that, the impact of defects at the Ag_2_BaTiSe_4_/MoSe_2_ and Ag_2_BaTiSe_4_/buffer interfaces are examined by varying it from 10^12^ to 10^20^ cm^−3^. The obtained results corresponding to the different layer parameters are also supported by the C–V, C–F, QE measurements, energy band diagrams, electric field, recombination rates, etc., extracted from SCAPS-1D. Finally, the effect of series resistance (R_S_), shunt resistance (R_Sh_), and operating temperature are studied for the optimized solar cells.

## Results and discussion

### Simulation of initial solar cells

The initial solar cells are designed with the solar cell structure of front contact/AZO/IZO/buffers/Ag_2_BaTiSe_4_/MoSe_2_/Mo/Glass as shown in Fig. 1a where new alkaline earth metal chalcogenides such as MgS, CaS, SrS, BaS and conventional CdS are used as buffers. They are simulated using the parameters listed in Tables 1 and 2. The solar cell parameters of the initial solar cells are listed in Table 3, and the corresponding J–V graphs are provided in Fig. 1b. To be brief, initial PCEs of 18.72%, 11.65%, 15.93%, 15.47%, and 14.99% are obtained for MgS, CaS, SrS, BaS, and CdS-based Ag_2_BaTiSe_4_ solar cells, respectively. The performance of these solar cells is further enhanced by optimizing the material parameters of buffers, Ag_2_BaTiSe_4_ and MoSe_2_, and tuning their interface properties as mentioned in the methodology, which can be seen in the following sections.

**Table 3:** Initial solar cell parameters of novel Ag_2_BaTiSe_4_ solar cells.

Solar cell structure	V_OC_ (V)	J_SC_ (mA cm^−2^)	FF (%)	PCE (%)
AZO/IZO/MgS/Ag_2_BaTiSe_4_/MoSe_2_/Mo	0.592	39.48	80.03	18.72
AZO/IZO/CaS/Ag_2_BaTiSe_4_/MoSe_2_/Mo	0.488	39.19	60.83	11.65
AZO/IZO/SrS/Ag_2_BaTiSe_4_/MoSe_2_/Mo	0.720	39.45	56.01	15.93
AZO/IZO/BaS/Ag_2_BaTiSe_4_/MoSe_2_/Mo	0.658	39.29	59.75	15.47
AZO/IZO/CdS/Ag_2_BaTiSe_4_/MoSe_2_/Mo	0.569	39.32	66.90	14.99

### Optimization of buffers

The buffer plays a vital role in thin-film solar cells. It develops a p–n junction with the absorber and reduces the pinhole effect and leakage current by terminating the interaction between the absorber and the window layers^62^. Thus, studying their properties to obtain a high PCE is crucial. Therefore, the critical parameters such as thickness, carrier concentration, and defect density of all buffers, namely MgS, CaS, SrS, BaS, and CdS, are varied from 0.05 to 0.2 µm, 10^12^ to 10^20^ cm^−3^, and 10^12^ to 10^20^ cm^−3^ respectively, to broadly investigate their impact on the performance of solar cells.

#### Effect of buffer thickness

**Figure 2:**
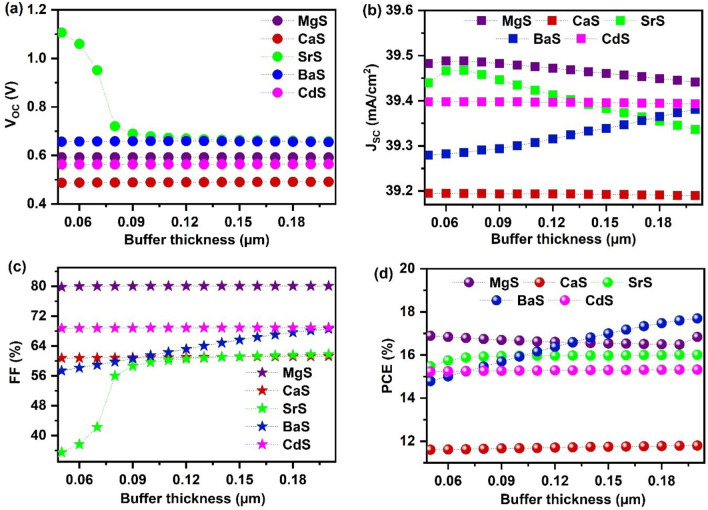
Effect of buffer’s thickness on (**a**) V_OC_ (**b**) J_SC_ (**c**) FF (**d**) PCE of novel Ag_2_BaTiSe_4_ based solar cells with diverse buffers.

The thickness of the buffer greatly influences the transportation of electrons from the absorber to the front contact. Therefore, it is crucial to determine the optimum thickness of each buffer to attain maximum solar cell performance. In this regard, we varied the thickness of MgS, CaS, SrS, BaS, and CdS from 0.05 to 0.2 µm, and the respective changes in V_OC_, J_SC_, FF, and PCE are displayed in Fig. 2. The corresponding J–V graphs are given in Fig. S1. It can be observed from Fig. 2a that the V_OC_ of SrS decreases from 1.1 to 0.67 V when the thickness is enhanced from 0.05 to 0.09 µm. The drastic decline may occur due to the reduced quasi-fermi level splitting in SrS-based solar cells with increasing thickness^63^. Nevertheless, it saturates beyond 0.09 µm. The V_OC_ of other buffers remains unaffected, indicating no change in the positions of energy bands and fermi levels in these solar cells corresponding to the buffer thickness^63^. On the other hand, the J_SC_ value increases from 39.27 to 39.38 mA cm^−2^ with an increase in BaS thickness. This is unusual because J_SC_ generally decreases with an increase in buffer thickness. It is suspected that this may be due to the characteristics of BaS, and further experimental study is required. In other cases, a small decrease in J_SC_ was observed, which could be attributed to the minute light absorption in buffers and the resulting reduction in charge carrier generation in the absorber^43^. Other researchers have noticed a minor change in J_SC_ due to an increase in buffer thickness, which is considered a constraint in numerical simulations^64–67^. Figure 2c shows that FF significantly increases from 35.47 to 58.64% till 0.09 µm in SrS-based solar cells and improves slightly to 61.8% on further increase to 0.2 µm. Similarly, it also enhances from 79.77 to 80.05%, 60.75 to 61.27%, 57.35 to 68.57%, and 68.71 to 68.93% in MgS, CaS, BaS, and CdS-based solar cells, respectively for the thickness range 0.050 to 0.2 µm. This increment originates from the reduction in the R_S_ of the solar cells at enhanced buffer thickness^68^. Consequently, PCE also increases in all solar cells (Fig. 2d). However, the improvement is minute, demonstrating that the impact of buffer thickness on the overall performance of all the solar cells is negligible. When making solar cells, the buffer thickness is a critical factor. If it's too thin (below 0.05 µm), it may not cover the entire substrate, causing a high leakage current and a poor p–n junction at the buffer/absorber interface. This, in turn, negatively impacts the spectral response of the solar cell^53,62,69,70^. On the other hand, if the buffer is too thick (over 0.1 µm), it leads to parasitic absorption, reducing the amount of charge carriers that reach the absorber, and reducing the built-in potential at the interface, which makes it harder to generate and collect charge carriers^62,70,71^. To achieve high PCE, it’s recommended to use a buffer thickness in the range of 0.05 to 0.1 µm for I_2_-II-IV-VI_4_ solar cells as found in the literature^16,51,55,58,71^. After considering all these factors, an optimal thickness of 0.080 µm is selected for the alkaline earth metal-based chalcogenide buffers, which falls within the suggested range.

#### Effect of buffer’s carrier concentration and defect density

**Figure 3:**
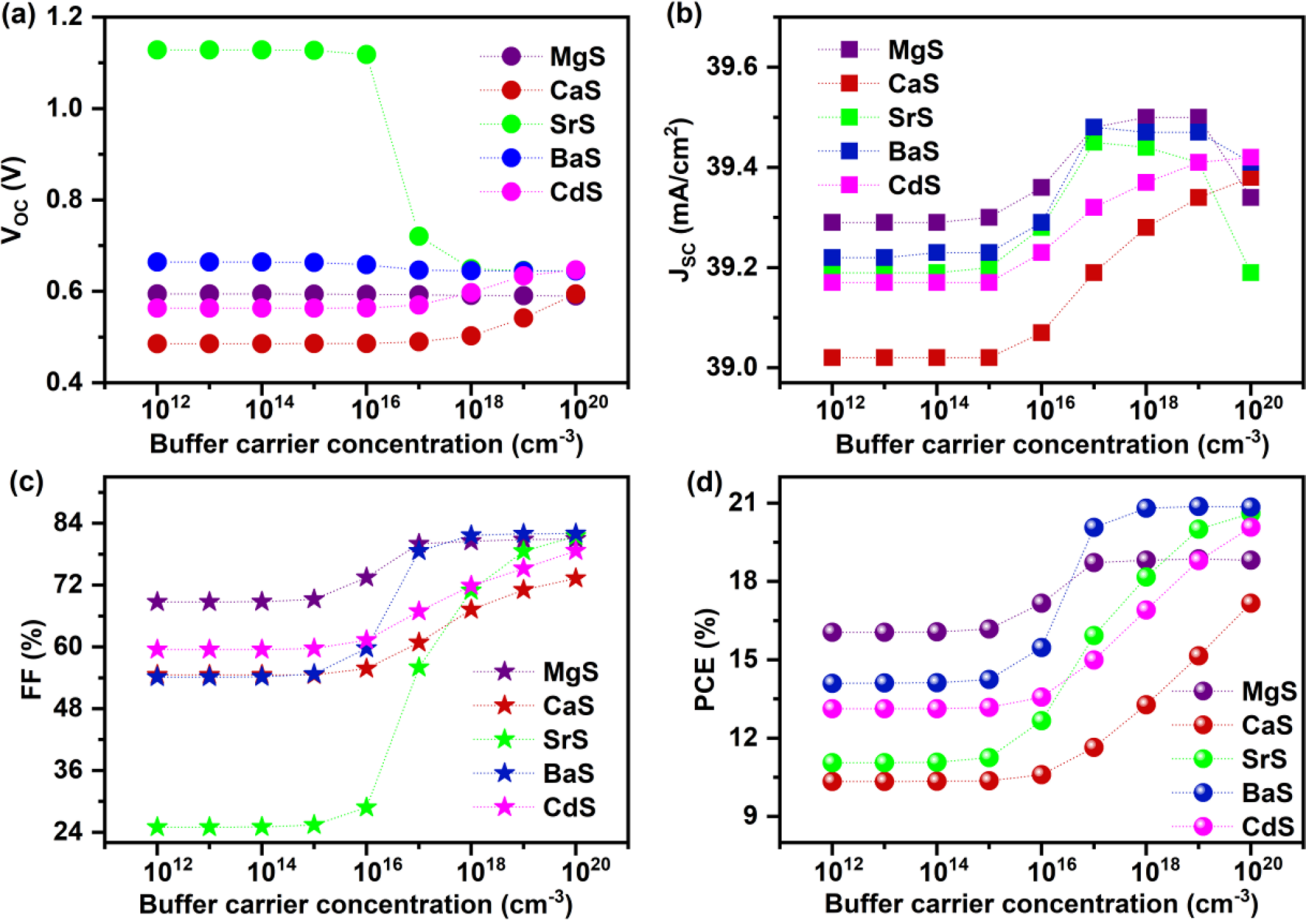
Effect of buffer’s carrier concentration on (**a**) V_OC_ (**b**) J_SC_ (**c**) FF (**d**) PCE of novel Ag_2_BaTiSe_4_ solar cells with diverse buffers.

The buffer’s carrier concentration strongly influences the interface properties between the absorber and buffer in solar cells^72^. Thus, to study its influence on solar cell parameters, each buffer’s carrier concentration (N_D buffer_) is varied from 10^12^ to 10^20^ cm^−3^ while fixing the absorber’s carrier concentration (N_A absorber_) at 10^15^ cm^−3^ as seen in Fig. 3. The respective J–V graphs are provided in Fig. S1. It can be observed that the V_OC_ of all solar cells stays unaltered till 10^16^ cm^−3^ and slightly increases for CaS and BaS-based solar cells while decreasing for SrS, MgS, and BaS-based solar cells beyond the mentioned value. It is well known that the V_OC_ of the solar cells increases with the splitting of electron and hole quasi-fermi levels, which is produced by the electrochemical potential difference of electrons and holes in each layer of solar cell^73^. In this view, the observed increase or decrease in V_OC_ can be attributed to the increment or reduction in the quasi-fermi level splitting in the respective solar cells with the increasing N_D buffer_. On the other hand, J_SC_ and FF values of all solar cells significantly improve above 10^15^ cm^−3^, leading to improved PCE. When N_D buffer_ > N_A absorber_, the concentration of electrons at the interface region rises, lowering the barrier height at the absorber/buffer interface. This subsequently elevates the built-in potential and conductivity of the solar cells, resulting in enhanced solar cell performance^72^. However, when the N_D buffer_ is increased beyond the optimum value, large electron–electron scattering occurs in solar cells, which hinders the carrier transportation, resulting in a slight decrease of J_SC_ and PCE for concentrations above 10^19^ cm^−3^ in MgS and BaS-based solar cells^74^. Thus, an optimum N_D buffer_ of 10^20^ cm^−3^ is chosen for CaS, SrS, and CdS buffers, while 10^19^ cm^−3^ is selected for MgS and BaS buffers to obtain the maximum solar cell performance.

**Figure 4:**
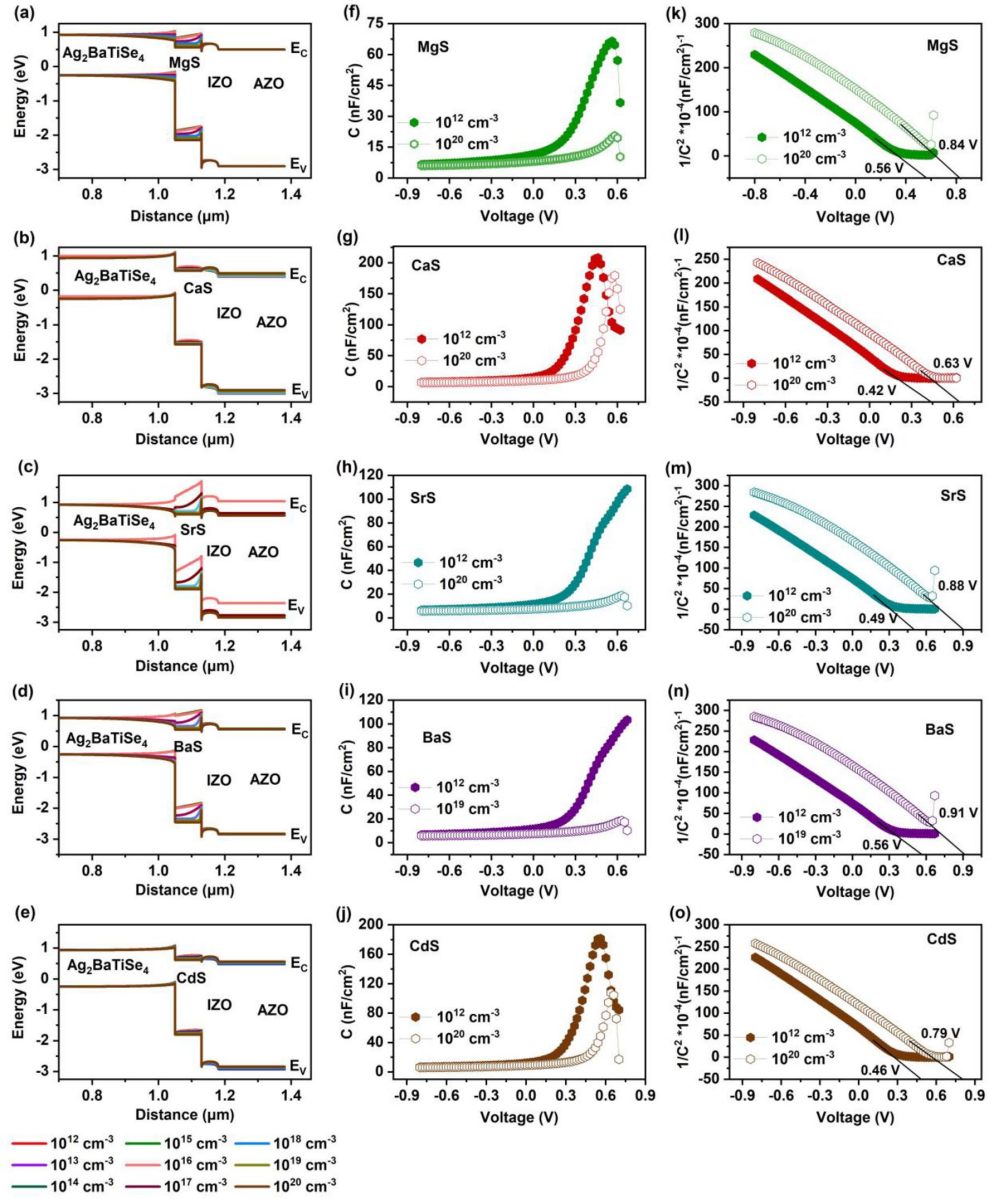
(**a-e**) Variation in energy band diagram of novel Ag_2_BaTiSe_4_ solar cells with diverse buffers as a function of N_D buffer_. (**f–j**) C–V and (**k–o**) Mott-Schottky plots with corresponding V_b_ at 10^12^ cm^−3^ and optimized N_D buffer_.

In general, N_D buffer_ primarily impacts the bending of energy bands at the absorber/buffer interface, facilitating the separation of charge carriers in solar cells^45^. Thus, to attain significant insights into its impact on band bending, the energy band diagram is extracted from SCAPS-1D as a function of N_D buffer,_ as shown in Fig. 4a–e, where E_C_ is the conduction band minimum, and E_V_ is the valence band maximum. It can be seen that when the N_D buffer_ is less than or equal to the N_A absorber_, no change is observed in the band alignment, attributing to the unaltered solar cell performance till 10^15^ cm^−3^. In addition, the barrier for electrons at the absorber/buffer and buffer/IZO interface is large. On the contrary, as the N_D buffer_ increases above the N_A absorber_, the E_C_ and E_V_ of the buffer move downwards, reducing the barrier at both interfaces and boosting the transport efficiency of charge carriers. This occurs because, at N_D buffer_ < N_A absorber_, the holes primarily occupy the absorber/buffer interface energy states, which act as a recombination center for the photogenerated electron, impeding its flow towards respective contact^72^. Moreover, as the electron concentration at the interface is very low, a large spike is formed at the absorber/buffer interface^75^. Nevertheless, for N_D buffer_ > N_A absorber_, the electrons predominantly occupy the lower density of states in the buffer’s conduction band and near interface states of absorber/buffer junction. This eventually improves the interaction and exchange potential between the charge carriers, resulting in a downward shift of energy bands. As a result, the barriers at the absorber/buffer and buffer/IZO interface shrink, enhancing the conductivity of the solar cells^45,76^. Furthermore, it could be noticed that the bending of buffer’s E_C_ and E_V_ is large in MgS, SrS, and BaS-based solar cells while it is less in CaS and CdS-based solar cells. This reveals that the influence of N_D buffer_ on the band bending of CaS and CdS-based solar cells is comparatively smaller than MgS, SrS, and BaS-based solar cells.

Impedance spectroscopy is a vital characterization technique to investigate the accumulation of charge carriers at the interface in solar cells^77^. Therefore, C–V measurements are performed between 10^12^ cm^−3^ and the optimum N_D buffer_ of each solar cell to analyze the accumulation of charge carriers at the absorber/buffer interface (Fig. 4f–j). All measurements are executed at 1 MHz frequency to address the repercussions of deep-level defects. From the C–V plots of all solar cells, it can be observed that the capacitance remains constant and has less values at lower voltages, corresponding to the depletion capacitance (C_DEP_), which originates from the depletion region at the absorber/buffer junction. Conversely, it is comparatively large at higher voltages. This behavior occurs because the depletion region shrinks as the voltage increases, resulting in a large accumulation of charge carriers at the interface. As a consequence, capacitance increases, which is termed as accumulation capacitance (C_ACC_)^78^. It can be noticed in Fig. 4f–j that the C_ACC_ of all solar cells at 10^12^ cm^−3^ is very high compared to the optimized N_D buffer_, denoting that the accumulation of charge carriers at the interfaces is intense at 10^12^ cm^−3^, which boosts the recombination in the bulk region. This is also evident in Fig. 4a–e, where the barrier for electrons at the Ag_2_BaTiSe_4_/buffer interface is large at 10^12^ cm^−3^. Thus, the photogenerated electrons in the absorber require more energy to cross the barriers and get collected at the front contact. As a result, they accumulate in the absorber and eventually recombine with the holes. Whereas, at an optimized N_D buffer_, the possibility for charge carrier accumulation decreases due to the reduction in the barrier, resulting in a fast collection of charge carriers without recombination. This has led to improved solar cell performance at an optimized N_D buffer_. Furthermore, in the C–V plot, we could notice that the voltage at which the capacitance begins to rise, shifts to a higher voltage for optimized N_D buffer_ in all solar cells. In other words, it can be said that C_DEP_ extends to higher voltage when the N_D buffer_ is increased, revealing that the depletion width at the p–n junction has been improved at a higher N_D buffer_. To witness it, the Mott Schottky (1/C^2^) graph is plotted from the C–V, and the built-in potential (V_b_) is determined from the intercept of the plot. It is clear from Fig. 4k–o that V_b_ has been drastically improved from 0.56 to 0.84 V, 0.42 to 0.63 V, 0.49 to 0.88 V, 0.56 to 0.91 V, 0.46 to 0.79 V for MgS, CaS, SrS, BaS, and CdS-based solar cells respectively, after optimizing the N_D buffer_. The corresponding depletion width (W) of each solar cell was calculated using the following equation^79^:

6 $${\text{Depletion}}\;{\text{width}} = \sqrt[2]{{\frac{{2{\upvarepsilon }_{0} {\upvarepsilon }_{{\text{S}}} {\text{V}}_{{\text{b}}} }}{{{\text{qN}}_{{\text{A absorber}}} }}}}$$

where ε_0_ is the dielectric permittivity of free space, ε_S_ is the dielectric constant, and q is the elementary charge. At 10^12^ cm^−3^, W is deduced to be 0.61 µm, 0.51 µm, 0.57 µm, 0.60 µm and 0.55 µm for MgS, CaS, SrS, BaS, and CdS-based solar cells respectively and improved to 0.74 µm, 0.64 µm, 0.76 µm, 0.77 µm and 0.72 µm at optimized N_D buffer_. It is essential to highlight that the W will extend towards the absorber as N_D buffer_ > N_A absorber_. Since most of the light absorption and charge carrier generation occurs in the depletion region along the absorber side, the increase in W boosts the amount of carrier generation in solar cells. Moreover, the elevation in V_b_ would hasten the charge carrier separation and improve their collection at the respective contacts^62^. Thus, final PCE of 18.81%, 17.17%, 20.6%, 20.85%, and 20.08% are obtained for MgS, CaS, SrS, BaS, and CdS-based solar cells, respectively, at the optimized N_D buffer_. The discussed research findings reveal N_D buffer's_ dominant role in enhancing solar cell performance.

**Figure 5:**
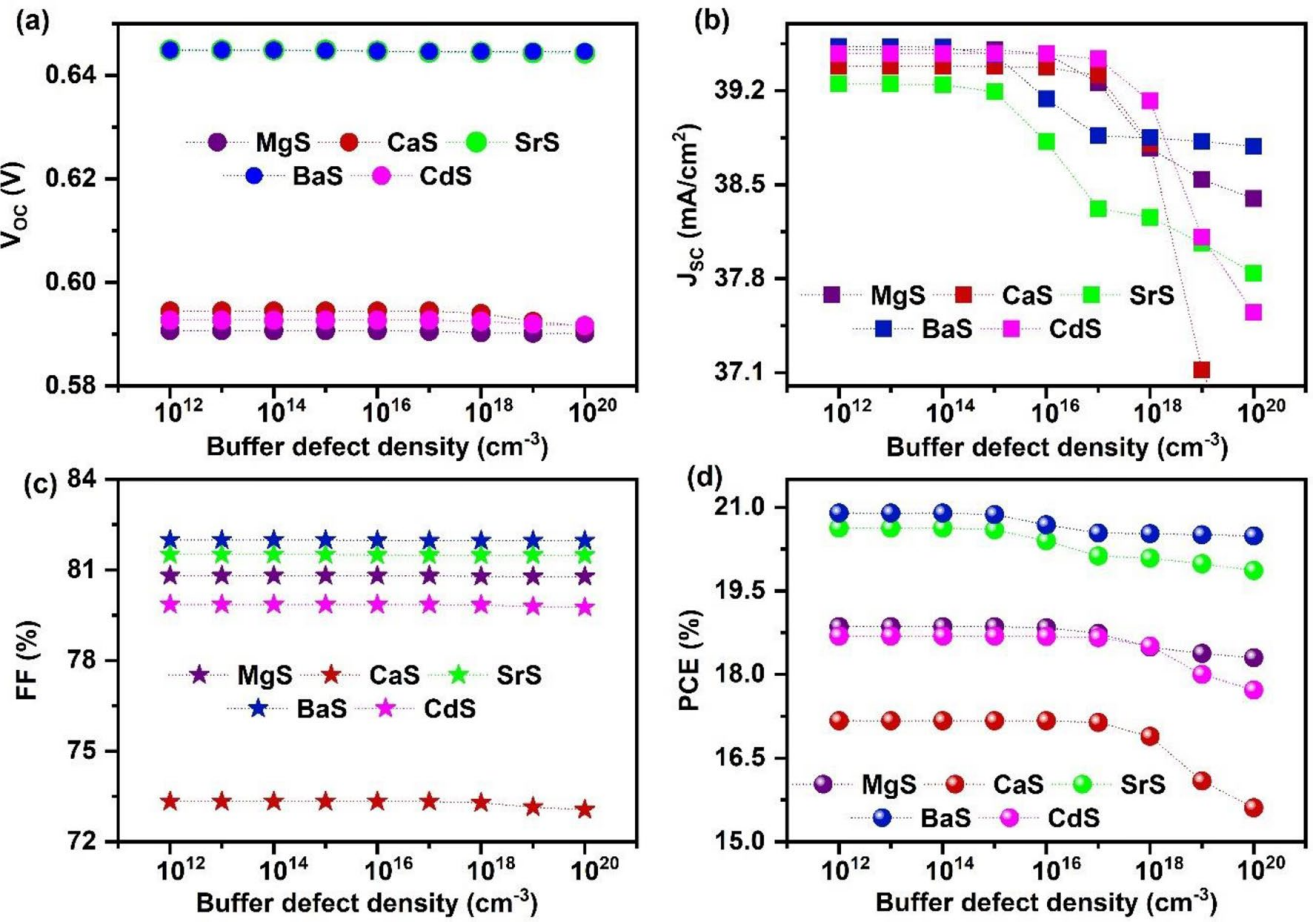
Effect of buffer’s defect density on (**a**) V_OC_ (**b**) J_SC_ (**c**) FF (**d**) PCE of novel Ag_2_BaTiSe_4_ solar cells with diverse buffers.

After that, the effect of defect density of each buffer is investigated by tuning it from 10^12^ to 10^20^ cm^−3^ (Fig. 5). The corresponding J–V graph is given in Fig. S1. All the photovoltaic parameters remain almost constant up to a specific range in all solar cells and slightly decrease to lower values with further increase in defects. In particular, PCE reduced from 18.86 to 18.3%, 17.17 to 15.61%, 20.63 to 19.87%, 20.9 to 20.49%, 18.69 to 17.72% for MgS, CaS, SrS, BaS, and CdS-based solar cells respectively, when the defect density is increased from 10^12^ to 10^20^ cm^−3^. The reduction in solar cell performance occurs because the rise in the defect states acts as traps for the charge carriers, which boosts the recombination rate in solar cells^80^. However, it can be noticed that the level of decrement is small, i.e., 0.56%, 1.56%, 0.76%, 0.41%, and 0.97% for MgS, CaS, SrS, BaS, and CdS-based solar cells. This indicates that the influence of the buffer’s defect density on the solar cell performance is negligible. Based on the results, an optimum defect density of 10^16^ cm^−3^ for MgS, 10^15^ cm^−3^ for BaS and SrS, and 10^17^ cm^-3^ for CdS and CaS are selected for further simulations.

### Absorber optimization

The absorber is the most critical layer in solar cells, as most charge carriers are generated here. Thus, the quality of the absorber is crucial to attain maximum solar cell performance. Therefore, the material parameters of Ag_2_BaTiSe_4,_ such as electron affinity, thickness, carrier concentration, and defect density, are varied from 4.1 to 4.7 eV, 0.1 to 2 µm, 10^12^ to 10^18^ cm^−3^ and 10^12^ to 10^20^ cm^−3^ respectively to understand their influence on the solar cell performance. The results of the Ag_2_BaTiSe_4_ optimization are elucidated in the following sections.

#### Effect of absorber’s electron affinity

**Figure 6:**
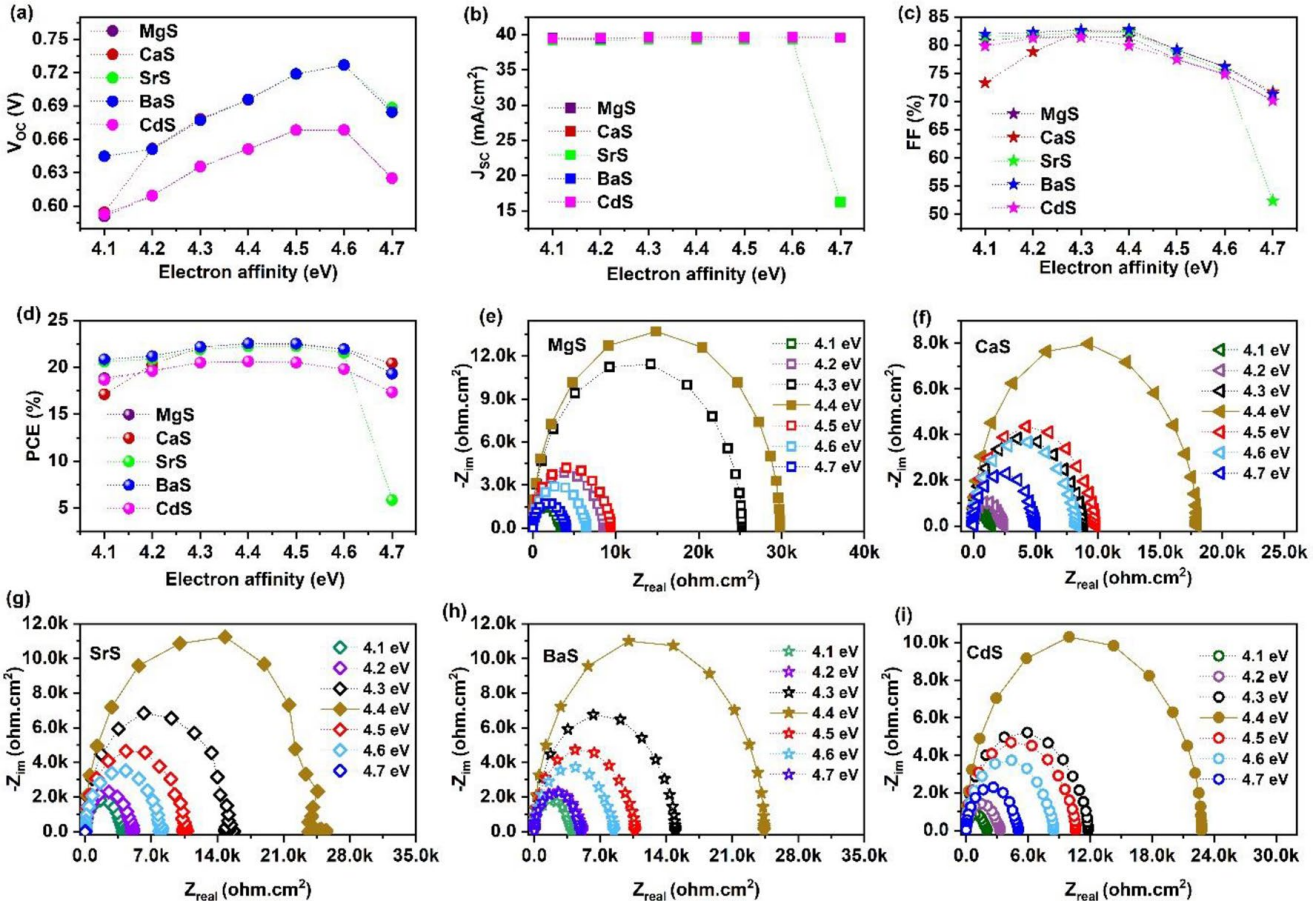
Effect of absorber’s electron affinity on (**a**) V_OC_ (**b**) J_SC_ (**c**) FF (**d**) PCE of novel Ag_2_BaTiSe_4_ solar cells with diverse buffers, (**e–i**) Nyquist plots as a function of absorber’s electron affinity.

Adjusting the energy band offsets at the absorber/buffer and MoSe_2_/absorber interface is essential to alleviate the formation of energy barriers and enhance the collection of charge carriers in solar cells^81^. This can be directly achieved by tuning the electron affinity of the absorber as it governs the energy band offsets at both interfaces. Herein, the electron affinity of Ag_2_BaTiSe_4_ is varied from 4.1 to 4.7 eV to identify the optimum energy band offset values in all solar cells. The variation in photovoltaic parameters as a function of electron affinity is displayed in Fig. 6a–d, and the corresponding J–V graphs are given in Fig. S2. The V_OC_ of all solar cells increases till 4.6 eV and reduces at 4.7 eV, while J_SC_ remains constant throughout the affinity range in all the solar cells except for SrS-based solar cells, where it drastically decreases after 4.6 eV. On the other hand, FF and PCE follow the same trend in all solar cells, and their maximum values are demonstrated at 4.4 eV. The affinity values above or below 4.4 eV degrade the solar cell performance. The obtained results can be clearly understood in light of variations in energy band offsets concerning the absorber’s electron affinity. High conduction band offset (CBO) and low valence band offset (VBO) are generally required at the MoSe_2_/absorber interface to restrict electrons and efficiently transport the photogenerated holes. Low CBO and high VBO are essential at the absorber/buffer interface to effectively collect electrons at the front contact^82^. Hence, CBO and VBO at both interfaces corresponding to each affinity value of absorber is calculated using the following formula^83^:

Absorber/buffer interface

7 $${\text{CBO }} = {\upchi }_{{{\text{absorber}}}} { }{-}{{ \chi }}_{{{\text{buffer}}}}$$

8 $${\text{VBO }} = { }\left[ {{\text{E}}_{{\text{G buffer}}} + {{ \chi }}_{{{\text{buffer}}}} } \right] - \left[ {{\text{E}}_{{\text{G absorber}}} + {{ \chi }}_{{{\text{absorber}}}} } \right]$$

MoSe_2_/absorber interface

9 $${\text{CBO }} = {\upchi }_{{{\text{absorber}}}} { }{-}{{ \chi }}_{{{\text{MoSe}}_{2} }}$$

10 $${\text{VBO }} = { }\left[ {{\text{E}}_{{{\text{G MoSe}}_{2} }} + {{ \chi }}_{{{\text{MoSe}}_{2} }} \left] { - { }} \right[{\text{E}}_{{\text{G absorber}}} + {{ \chi }}_{{{\text{absorber}}}} } \right]$$

where ${\upchi }_{{{\text{MoSe}}_{2} }}$, ${\upchi }_{{{\text{buffer}}}}$, ${\upchi }_{{{\text{absorber}}}}$ and ${\text{E}}_{{{\text{G MoSe}}_{2} }}$, ${\text{E}}_{{\text{G buffer}}}$, ${\text{E}}_{{\text{G absorber}}}$ are the affinity and bandgap of MoSe_2_, buffer, and absorber respectively. The calculated CBO and VBO of all solar cells at both interfaces are explicitly provided in Table 4. On analyzing the MoSe_2_/absorber interface, VBO holds positive and negative values with the absorber’s electron affinity variation. At positive VBO, a spike is created at the MoSe_2_/absorber interface, which hinders the diffusion of holes from the absorber to MoSe_2_. In the case of negative VBO, the photogenerated holes in the absorber have a cliff-like barrier that boosts the collection of holes at the back contact. However, a large cliff elevates the accumulation of holes in MoSe_2_, which enhances the recombination at the back contact, deteriorating the solar cell performance^84^. Thus, the optimum VBO at the MoSe_2_/absorber interface is identified to be − 0.06 eV to demonstrate maximum PCE. Similarly, two conditions are observed at the absorber/buffer interface^84^: (1) When the affinity of the absorber is less than the buffer, negative CBO is obtained, representing cliff formation. (2) Positive CBO is attained when it is higher than the buffer, leading to spike formation at the interface.

**Table 4:** Electron affinity of absorber with the corresponding CBO and VBO at the interfaces of novel Ag_2_BaTiSe_4_ solar cells with diverse buffers.

Affinity (eV)	Absorber/buffer interface										Absorber/MoSe_2_ interface	
	MgS		SrS		BaS		CaS		CdS			
	CBO (eV)	VBO (eV)	CBO (eV)	VBO (eV)	CBO (eV)	VBO (eV)	CBO (eV)	VBO (eV)	CBO (eV)	VBO (eV)	CBO (eV)	VBO (eV)
4.1	− 0.2	1.72	0.1	1.22	− 0.05	1.87	− 0.5	1.46	− 0.4	1.64	− 0.02	0.24
4.2	− 0.1	1.62	0.2	1.12	0.05	1.77	− 0.4	1.36	− 0.3	1.54	0.08	0.14
4.3	0	1.52	0.3	1.02	0.15	1.67	− 0.3	1.26	− 0.2	1.44	0.18	0.04
**4.4**	**0.1**	**1.42**	**0.4**	**0.92**	**0.25**	**1.57**	− **0.2**	**1.16**	− **0.1**	**1.34**	**0.28**	− **0.06**
4.5	0.2	1.32	0.5	0.82	0.35	1.47	− 0.1	1.06	0	1.24	0.38	− 0.16
4.6	0.3	1.22	0.6	0.72	0.45	1.37	0	0.96	0.1	1.14	0.48	− 0.26
4.7	0.4	1.12	0.7	0.62	0.55	1.27	0.1	0.86	0.2	1.04	0.58	− 0.36

Optimum values are in [bold].

It has been commonly believed for a long time that a cliff at the absorber/buffer interface is beneficial for solar cells, as the separation and extraction of the charge carriers are not constrained while it is restricted by spikes due to the formation of barrier at the interface^85^. Later, the development of simulation tools and experimental results proved that a moderate spike-like barrier is also advantageous for solar cells as it creates strong V_b_ at the interface, enhancing the carrier collection at the contacts^86,87^. Moreover, in some cases, a large cliff-like barrier has also been observed, which leads to charge carrier accumulation at the interface due to the weak potential barrier, improving the interfacial recombination and thereby affecting the solar cell performance^88^. Thus, it is important to mention that the ideal type of barrier (either spike or cliff) required at the absorber/buffer interface and optimum values of the barrier height in solar cells are scattered in the literature, revealing that it primarily depends on the adopted material system^75,87,89^. The results show that CaS, CdS-based solar cells require a cliff-like barrier with a height of − 0.2 eV and − 0.1 eV to attain maximum solar cell performance. Whereas the spikes of 0.1 eV, 0.4 eV, and 0.25 eV are essential in MgS, SrS, and BaS-based solar cells. The positive or negative CBO above or below the mentioned values would degrade the solar cell performance due to the unfavorable band alignment at the absorber/buffer interface. Furthermore, the obtained results exhibit that the difference in the type of barrier and their respective barrier height mainly stems from the material characteristics of the buffer, as the other layers in these solar cells are similar, revealing the dominant role of buffer properties for the efficient transportation of charge carriers. It has also been reported elsewhere that buffer’s parameters, such as carrier concentration, the effective density of states, interface defect states, etc., determine the barrier height at the interface, thus confirming the demonstrated results^75^. Moreover, the optimum VBO and CBO calculated at the absorber/buffer and MoSe_2_/absorber interface, respectively, are also ideal for restricting the holes and electrons in the respective interfaces. Hence, the electron affinity of 4.4 eV is required in Ag_2_BaTiSe_4_ to achieve high PCE.

In addition, to gain a deep understanding of the variations in the transportation and recombination of charge carriers corresponding to the absorber’s electron affinity, Nyquist plots are plotted from C-F measurements in all solar cells, as shown in Fig. 6e–i. Generally, the Nyquist plot of solar cells consists of two semi-circles at separate frequency regions. The semi-circle at the low-frequency range denotes the recombination resistance (R_rec_) at the absorber/buffer interface, while the high-frequency semicircle signifies the hole transfer resistance at the MoSe_2_/absorber interface^90^. Interestingly, a single semi-circle is observed in all these solar cells in the whole frequency range. In addition, the semicircle obtained at the optimum electron affinity, i.e., 4.4 eV, is larger than the other affinity values in all solar cells. This indicates that the obtained semicircle represents the R_rec_. The absence of high-frequency semicircles reveals no hole transfer resistance in these solar cells. As discussed above, the large semicircle at 4.4 eV displays that the solar cells have high R_rec_ at the optimum electron affinity, occurring due to the proper CBO and VBO at the interfaces of the absorber and transporting layers. The shrinkage in the semicircle for the affinity values above or below 4.4 eV happens due to the surging recombination rate of charge carriers resulting from improper interface barriers. From the above discussions, it is apparent that proper CBO and VBO at the absorber/buffer and MoSe_2_/absorber interface are essential to effectively transport the photogenerated charge carriers to the contacts without recombination.

#### Effect of absorber’s thickness

**Figure 7:**
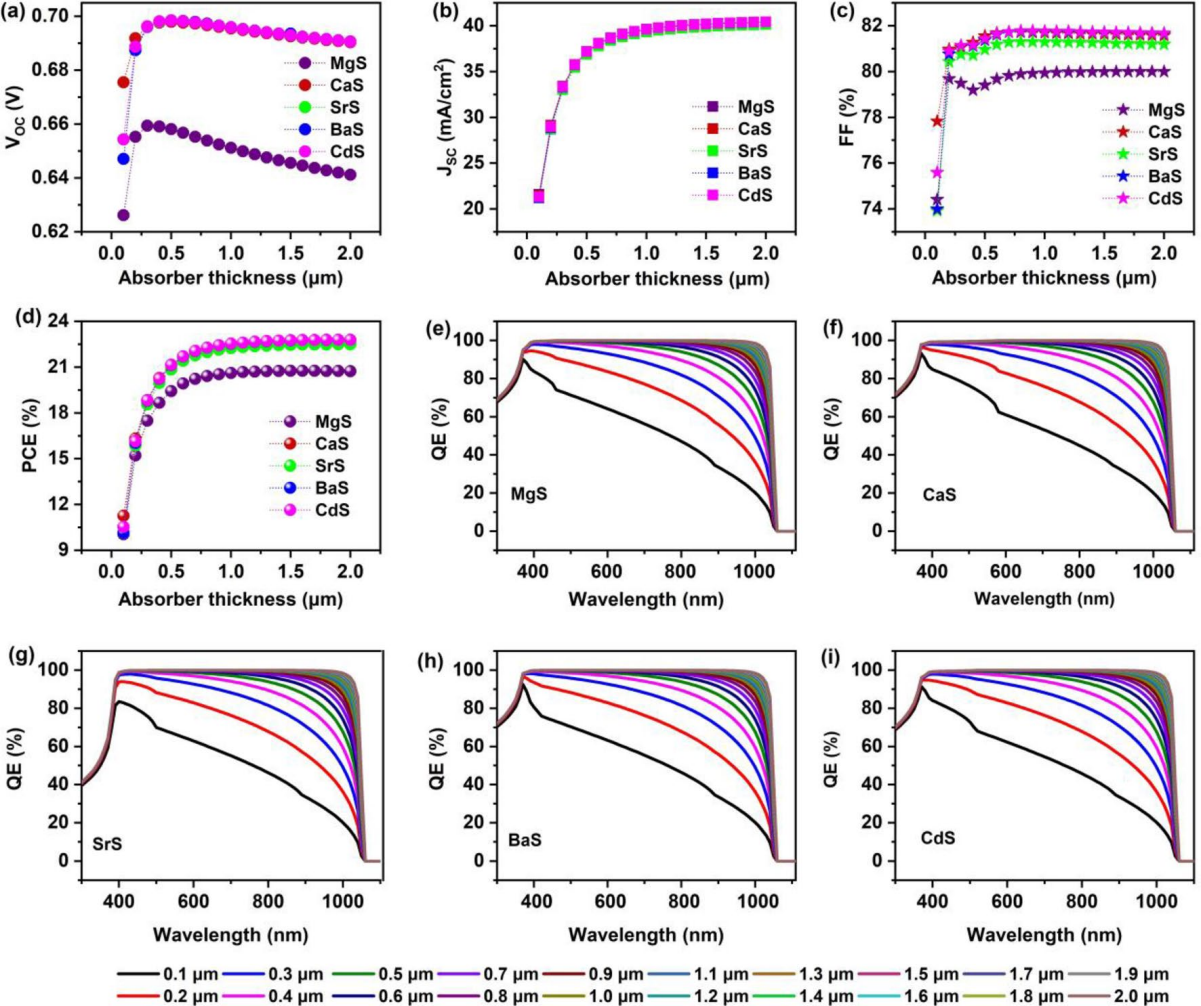
Effect of absorber’s thickness on (**a**) V_OC_ (**b**) J_SC_ (**c**) FF (**d**) PCE (**e-i**) QE of novel Ag_2_BaTiSe_4_ solar cells with diverse buffers.

The thickness of the absorber plays a vital role in understanding the performance of solar cells. Thus, the thickness of Ag_2_BaTiSe_4_ is varied from 0.1 to 2 µm to identify the optimum value. Figure 7a–d displays the variations in V_OC_, J_SC_, FF, and PCE as a function of absorber thickness. The respective J–V graphs are given in Fig. S2. It can be noticed that the V_OC_ of all solar cells improves till ~ 0.3 µm and slightly decreases after the mentioned value. The initial improvement is attributed to enhanced quasi-fermi level splitting with the large generation of charge carriers whereas its reduction originates from the elevating dark saturation current and recombination rate with the thickness^91^. In addition, FF rises till a certain thickness range and saturates in all the solar cells, occurring due to the enhanced R_S_ in a solar cell for thicker absorber^91^. On the other hand, when the thickness is increased from 0.1 to 2 µm, J_SC_ drastically improves from ~ 21 to ~ 40 mA cm^−2^ in all solar cells. This has subsequently elevated PCE from 10.05 to 20.74%, 11.27 to 22.75%, 10.13 to 22.75%, 10.13 to 22.5%, 10.15 to 22.79%, and 10.54 to 22.79% in MgS, CaS, SrS, BaS and CdS-based solar cells respectively. This happens because when the absorber is very thin, photons of longer wavelengths from the sun are not absorbed, and most of the light is transmitted. This results in the poor generation of charge carriers in solar cells due to low performance. As the thickness increases, the photon absorption in solar cells is enhanced, elevating the generation rate of charge carriers^92^. This has led to a steep rise in solar cell performance. However, we could see that J_SC_ significantly improves till 1 µm and saturates beyond the mentioned value. A similar trend has also been observed in PCE. In particular, when the thickness is enhanced from 0.1 to 1 µm, the increment in PCE is about 10.57%, 11.23%, 12.12%, 12.38%, and 12% for MgS, CaS, SrS, BaS, and CdS-based solar cells respectively. While it increased by just ~ 0.25% in all the solar cells when the thickness was extended 1–2 µm. When the absorber is too thick, the generated charge carriers must travel long distances to reach the respective contacts. Thus, the majority of them tend to recombine due to shorter diffusion lengths than the absorber thickness, causing saturated solar cell performance^93^. This is also evident in QE measurements (Fig. 7e–i), where the absorption increases by ~ 34% in all the solar cells for the thickness range of 0.1 to 1 µm. Whereas it improves by ~ 1.5% on further increment to 2 µm. In I_2_-II-IV-VI_4_ solar cells, experiments have shown that an absorber thickness of approximately 1 µm is ideal for majority photon absorption due to its high absorption coefficient (~ 10^5^ cm^−1^)^54,94–96^. For example, when the thickness of CZTS was adjusted from 0.5 to 2 µm, the PCE gradually increased up to 1 µm and then stabilized beyond that. QE measurements revealed that the collection depth of minority carriers ranged from 0.75 to 1 µm, indicating that recombination was high for thicknesses greater than 1 µm. Conversely, for absorber thicknesses less than 0.75 µm, there were high collection losses of charge carriers in the solar cells. Therefore, the optimal thickness for the CZTS absorber was determined to be around 1 µm for achieving high PCE^97^. Our simulation results were compared with the experiments, and we concluded that a thickness of 1 µm for Ag_2_BaTiSe_4_ would be sufficient to achieve high solar cell performance.

#### Effect of absorber’s carrier concentration

**Figure 8:**
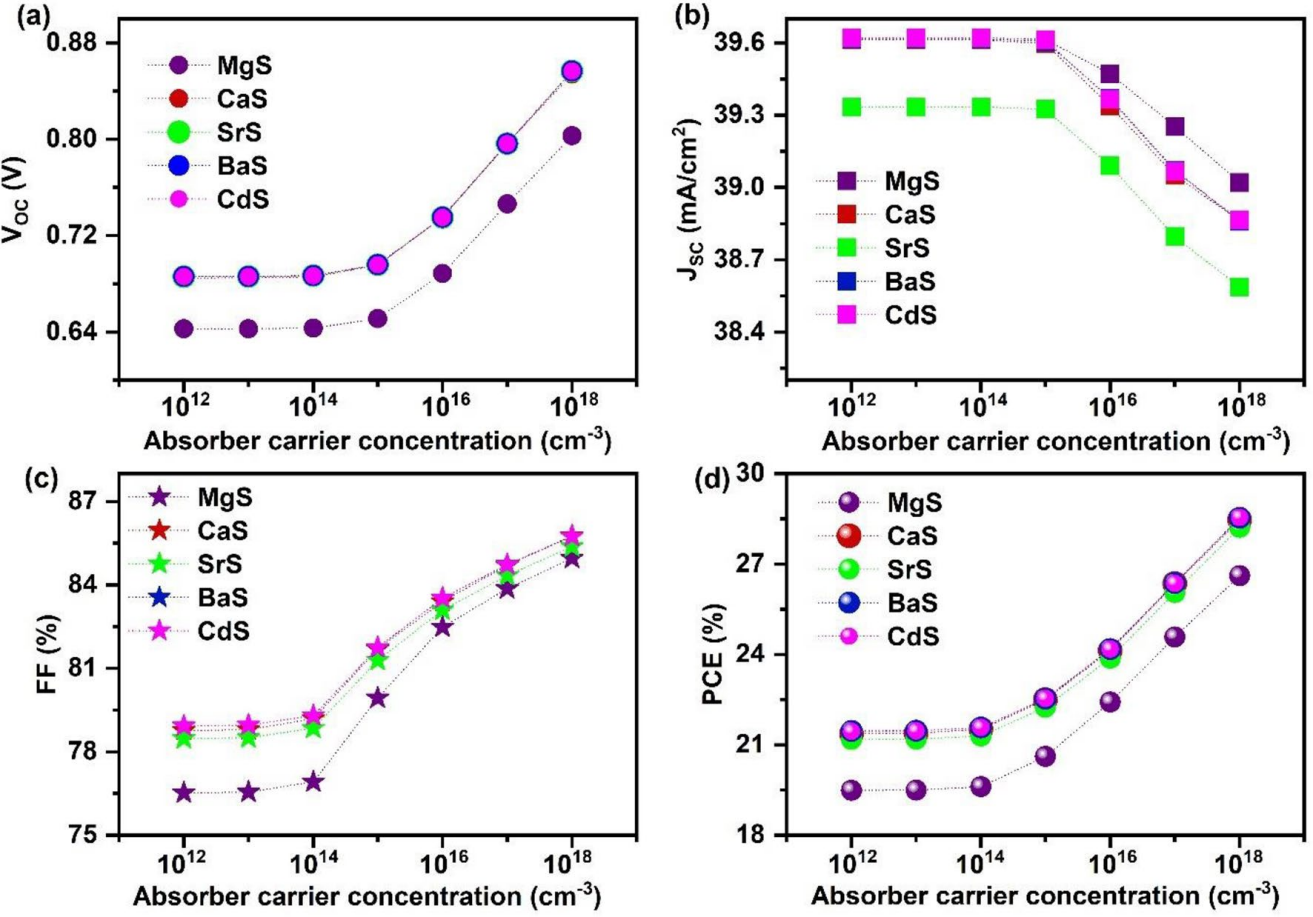
Effect of absorber’s carrier concentration on (**a**) V_OC_ (**b**) J_SC_ (**c**) FF (**d**) PCE of novel Ag_2_BaTiSe_4_ solar cells with diverse buffers.

The N_A absorber_ modifies its electrical conductivity and determines the charge separation efficiency of the solar cells^98^. According to the Mott criterion, the maximum carrier concentration limit of CZTS is 10^18^ cm^−3^. Beyond this, it degenerates and loses its semiconducting property, adversely affecting the J_SC_ of solar cells^99^. Thus, taking insights from the parent material, the carrier concentration of Ag_2_BaTiSe_4_ is varied from 10^12^ to 10^18^ cm^−3^ in all solar cells. The changes in the solar cell parameters concerning the N_A absorber_ are displayed in Fig. 8. The J–V graphs are provided in Fig. S3. It can be seen that the V_OC_ and FF of all solar cells are unchanged till 10^14^ cm^−3^, and then it drastically increases with the N_A absorber_.

**Figure 9:**
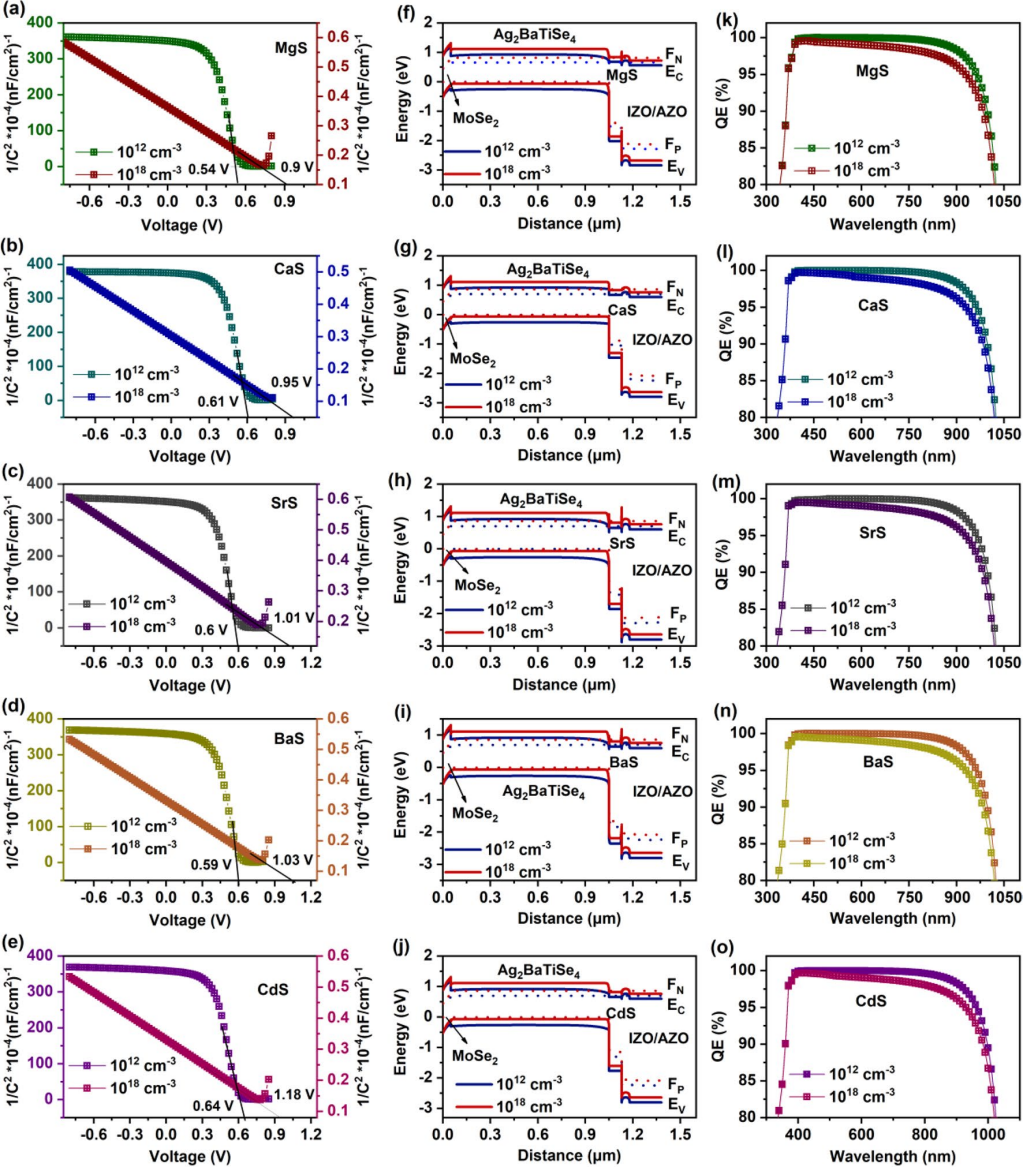
(**a–e**) Mott-Schottky plots with respective V_b_, (**f–j**) Energy band diagram and (**k–o**) Changes in QE (magnified image) at absorber’s carrier concentration of 10^12^ and 10^18^ cm^−3^ in novel Ag_2_BaTiSe_4_ solar cells with diverse buffers. The simplified illustration of QE is provided in Fig. S4.

In general, the improvement in the N_A absorber_ amplifies the V_b_ at the interface, promoting the separation efficiency of photogenerated charge carriers^100^. Mott-Schottky plots for 10^12^ and 10^18^ cm^−3^ and their corresponding V_b_ are shown in Fig. 9a–e. When N_A absorber_ is 10^12^ cm^−3^, V_b_ of MgS, CaS, SrS, BaS, and CdS-based solar cells are identified to be 0.54 V, 0.61 V, 0.6 V, 0.59 V and 0.64 V respectively which are then increased to 0.9 V, 0.95 V, 1.01 V, 1.03 V and 1.18 V at 10^18^ cm^−3^. Thus, the attained increment in V_b_ assists in efficiently separating and collecting the generated charge carriers at the respective electrodes without recombination, leading to a drastic rise in V_OC_ and FF. In addition, the increase in N_A absorber_ modifies the energy band alignment and enhances the quasi-fermi level splitting, boosting the V_OC_ and overall performance of solar cells. Therefore, to witness it, energy band diagrams for 10^12^ and 10^18^ cm^−3^ are extracted from SCAPS-1D in all solar cells (Fig. 9f–j). When N_A_ is increased from 10^12^ to 10^18^ cm^−3^, the energy bands of all the layers in the solar cell shift upwards, such that the E_V_ of the absorber moves closer to the hole quasi-fermi level (F_P_). This strongly elevates the conductivity of solar cells, leading to improved solar cell performance. Notably, no change is observed in the position of F_P_ along Ag_2_BaTiSe_4_ and MoSe_2_, whereas the electron quasi-fermi level (F_N_) shifts upward along with the energy bands with increasing N_A absorber_, disclosing the increment in the splitting of quasi-fermi levels, which directly improves the V_OC_ of solar cells. Contrastingly, J_SC_ remains constant till 10^15^ cm^−3^ and decreases with a further increase in the N_A absorber_. This happens because as the N_A absorber_ increases, the width of the depletion region decreases along the absorber region while improving towards the buffer, which reduces the light absorption in solar cells^45^. This can also be evidenced in Fig. 9k–o, where QE is performed between 10^12^ and 10^18^ cm^−3^ in all solar cells. The absorption slightly decreases by 1.18%, 1.14%, 1.2%, 1.17%, and 1.16% in MgS, CaS, SrS, BaS, and CdS-based solar cells, causing a reduction in J_SC_. In addition, the possibility of charge carrier generation in the quasi-neutral region of the absorber increases with a decrement of W in the absorber, and the charge carriers generated far from the junction have to diffuse through W to reach the contacts. Some of them with insufficient diffusion length and lifetime may recombine, diminishing the J_SC_ of solar cells^100^. However, when the N_A absorber_ is increased from 10^12^ to 10^18^ cm^−3^, PCE drastically rises from 19.49 to 26.62%, 21.37 to 28.47%, 21.18 to 28.22%, 21.46 to 28.54% and 21.45 to 28.55% for MgS, CaS, SrS, BaS, and CdS-based solar cells respectively which mainly comes from the combined improvement of V_OC_ and FF values while the demise in J_SC_ does not affect the overall performance of the solar cells.

#### Effect of absorber’s defect density

**Figure 10:**
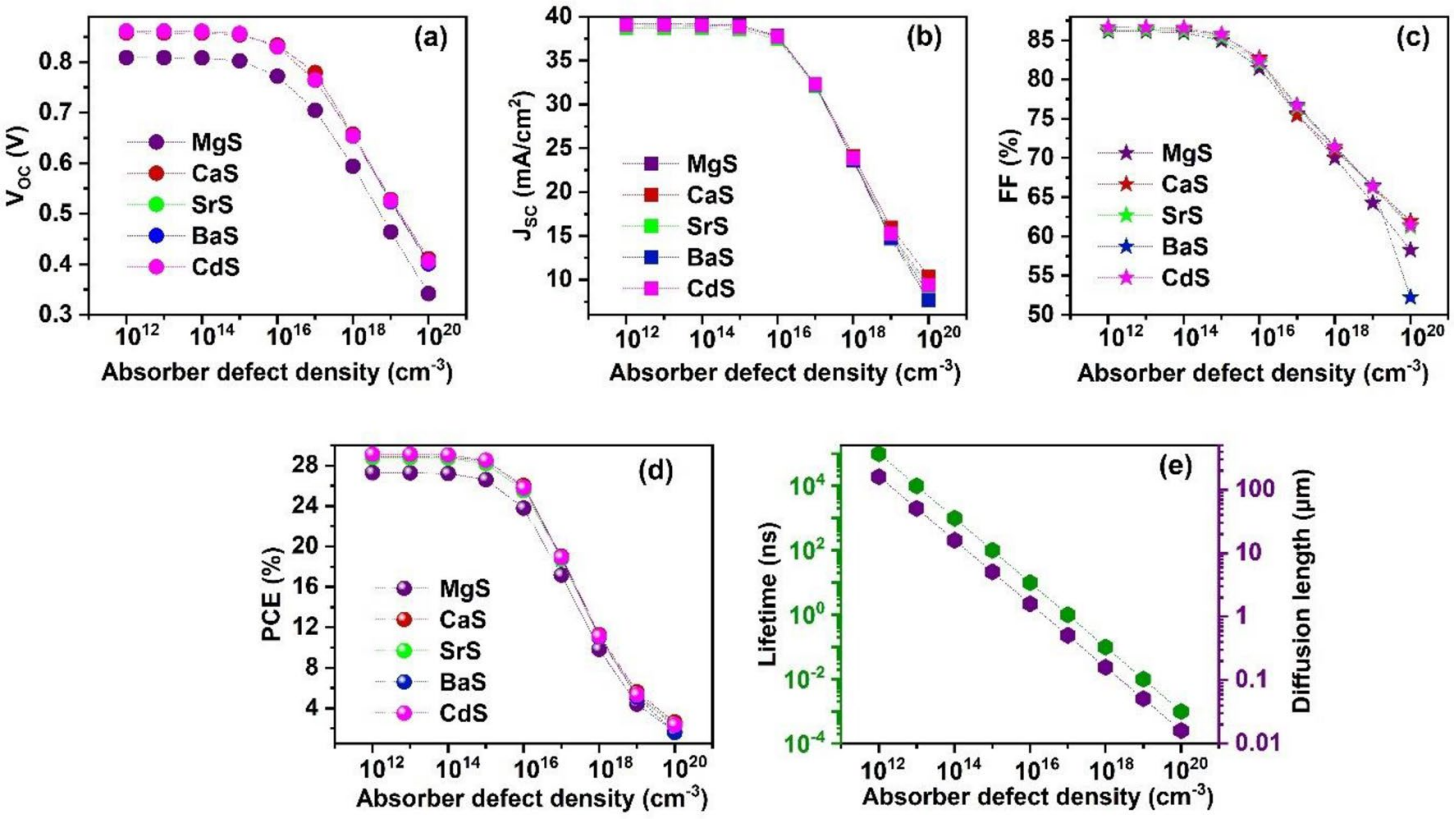
Effect of absorber’s defect density on (**a**) V_OC_ (**b**) J_SC_ (**c**) FF (**d**) PCE and (**e**) diffusion length and lifetime of charge carriers of novel Ag_2_BaTiSe_4_ solar cells with diverse buffers.

The optoelectronic properties of solar cells are adversely affected by defects in the material system^81^. Therefore, the influence of the absorber’s defect density on the photovoltaic parameters is examined by tuning it from 10^12^ to 10^20^ cm^−3^ in all solar cells. Figure 10a–d shows the changes in V_OC_, J_SC_, FF, and PCE as a function of absorber defect density, and the respective J–V is displayed in Fig. S3. All the solar cell parameters are almost unaltered till 10^15^ cm^−3^ and dramatically falls on further increase in defect density. To be specific, when defect density is improved from 10^12^ to 10^20^ cm^−3^, PCE sharply declines from 27.3 to 1.68%, 28.9 to 2.64%, 28.8 to 2.22%, 29.13 to 1.6% and 29.13 to 2.33% for MgS, CaS, SrS, BaS, and CdS-based solar cells respectively. The massive decline occurs due to the increase in the recombination sites at the path of photogenerated charge carriers with increasing defects, which shortens their diffusion length and lifetime^101^. Generally, the minority carrier lifetime ($\tau )$ is calculated using Eq. (11)^102^

11 $$\tau = \frac{1}{{\sigma v_{th} N_{t} }}$$

Here $\sigma$ is the capture cross-section of the charge carrier, v_th_ is the thermal velocity, and N_t_ is the defect density.

The diffusion length of charge carriers is given as

12 $$L = \sqrt {D\tau }$$

where L is the minority carrier diffusion length and D is the diffusion coefficient. Herein, the L and τ are acquired from SCAPS-1D for the varying defect density of the absorber with a thickness of 1 µm (Fig. 10e). When the defect density is raised from 10^12^ to 10^20^ cm^−3^, the τ considerably decreased from 10^5^ to 10^–3^ ns and their L drastically reduced from 160 µm to 0.016 µm. On comparing the obtained solar cell parameters with Fig. 10e, it can be observed that the PCE almost remains unaffected till L of 5.1 µm, slightly decreases for L = 1.6 µm while firmly falls for L ≥ 0.5 µm, confirming that L less than the thickness of the absorber adversely affects the solar cell performance. Moreover, the observed decrement in L and τ elevates the recombination rate of charge carriers, leading to poor solar cell performance.

**Figure 11:**
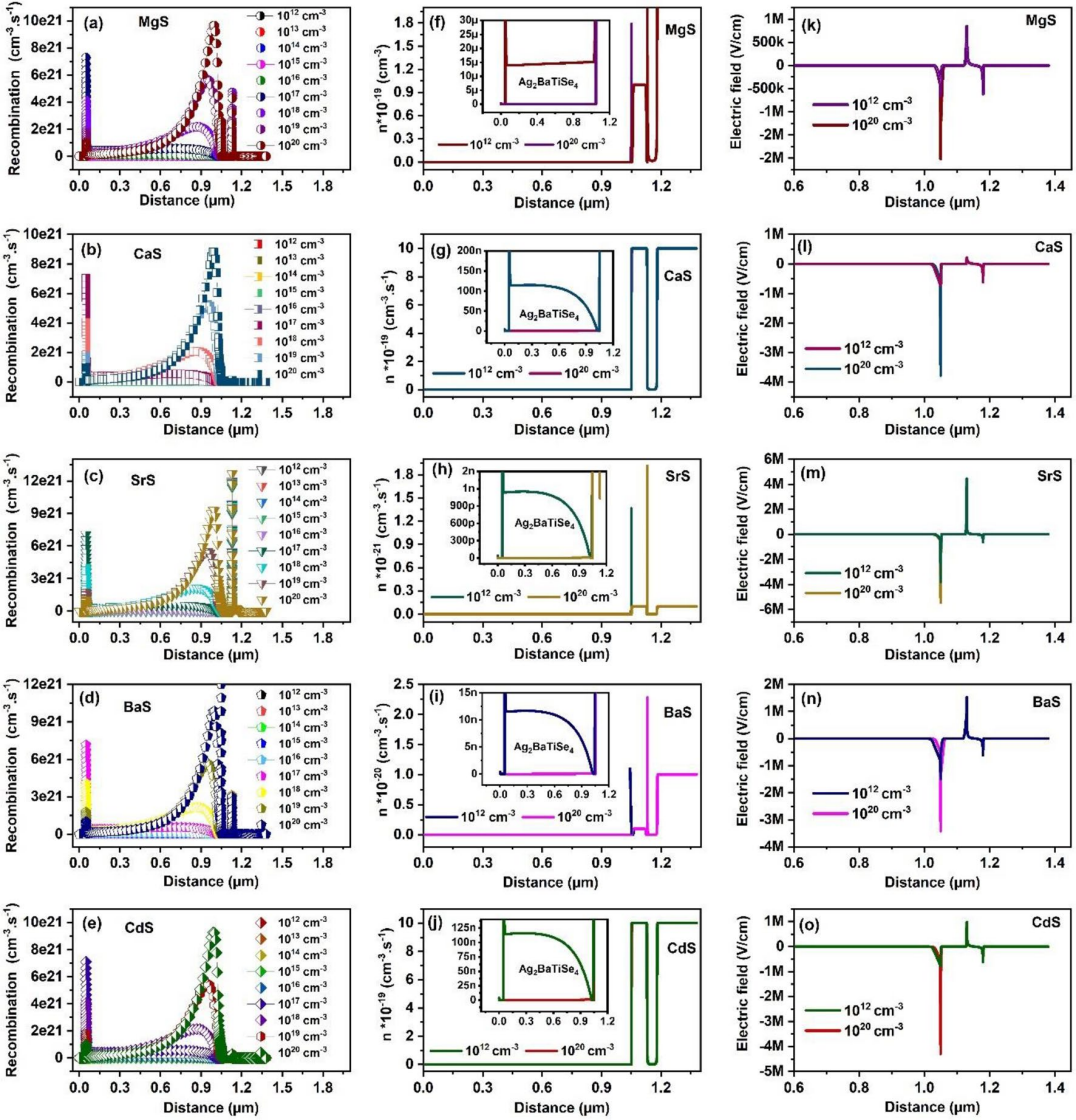
(**a–e**) Recombination rate as a function of defect density of absorber. (**f–j**) Change in the solar cells’ density of electrons ‘n’ with the increasing defect density. The inset displays the variation in ‘n’ along the absorber region. (**k–o**) Electric field corresponding to defect density of absorber.

The recombination rate concerning the increasing defect density in all solar cells is shown in Fig. 11a–e. As the defect density increases, the recombination rate shoots up in the absorber region near the absorber/buffer junction of all solar cells, giving rise to deterioration in solar cell performance. Interestingly, the recombination at the absorber/MoSe_2_ interface decreases with defect density. This occurs because, as the recombination in the absorber region increases, the density of photogenerated carriers drastically reduces in the entire bulk region of the absorber and the near interface regions. This subsequently declines the number of charge carriers reaching the electrodes, reducing the recombination rate at the absorber/MoSe_2_ interface. This is also evident from Fig. 11f–j where the variation in density of minority carriers (electrons ‘n’) in the absorber region for the defect density 10^12^ and 10^20^ cm^−3^ of all solar cells are explicitly displayed. It can be seen that the density of electrons in the absorber region becomes negligible at 10^20^ cm^−3^, resulting from the hiking recombination rate in the absorber. In addition, electrons at the absorber/buffer interface of MgS, SrS, and BaS-based solar cells are diminished at 10^20^ cm^−3^, indicating that there is sharp recombination at the absorber/buffer interface of these solar cells as witnessed in Fig. 11a, c, d. Furthermore, the electric field at the interface of the absorber and buffer is minimized with increasing defects, as shown in Fig. 11k–o. Thus, the separation and collection of charge carriers are considerably affected, worsening the performance of solar cells. Overall, the results show that the defect density rise greatly affects the L and τ of charge carriers, increasing the recombination rate in solar cells. Consequently, the density of minority carriers and built-in electric field at the p–n junction declined, drastically reducing the solar cells' overall performance. In light of the obtained results, 10^15^ cm^−3^ is selected as the optimum defect density of Ag_2_BaTiSe_4_ for all solar cells.

**Figure 12:**
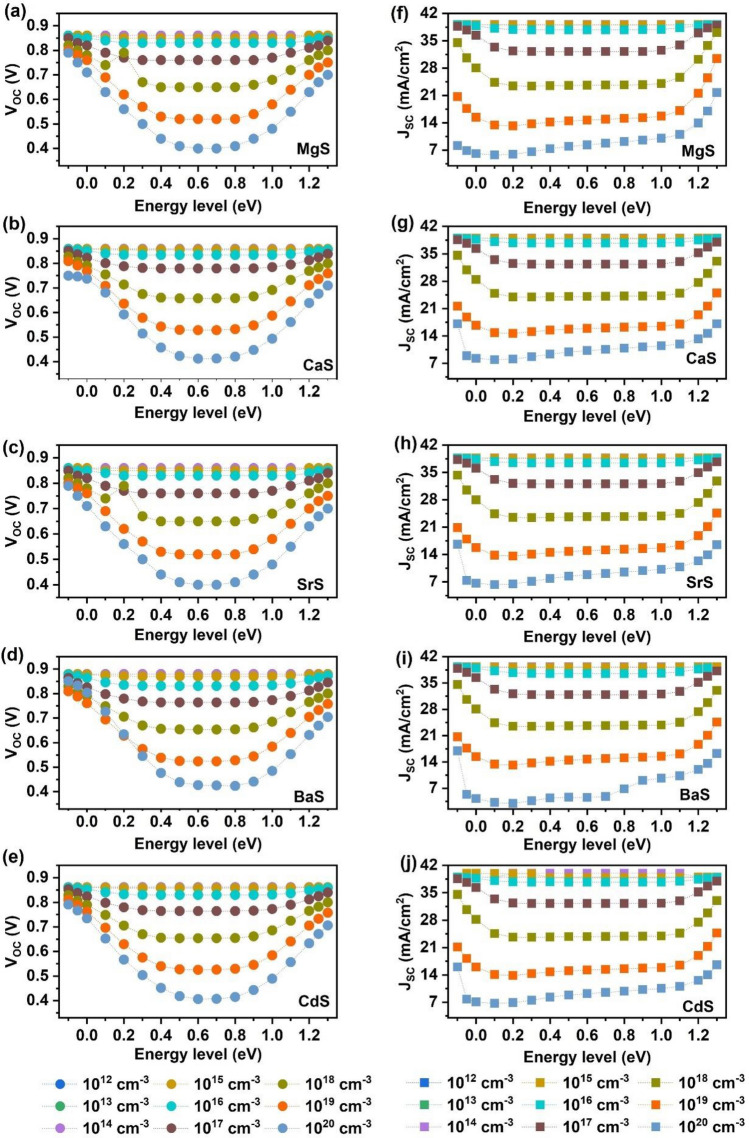
Effect of absorber’s defect energy level and density on (**a–e**) V_OC_ and (**f–j**) J_SC_ of novel Ag_2_BaTiSe_4_ solar cells with diverse buffers.

**Figure 13:**
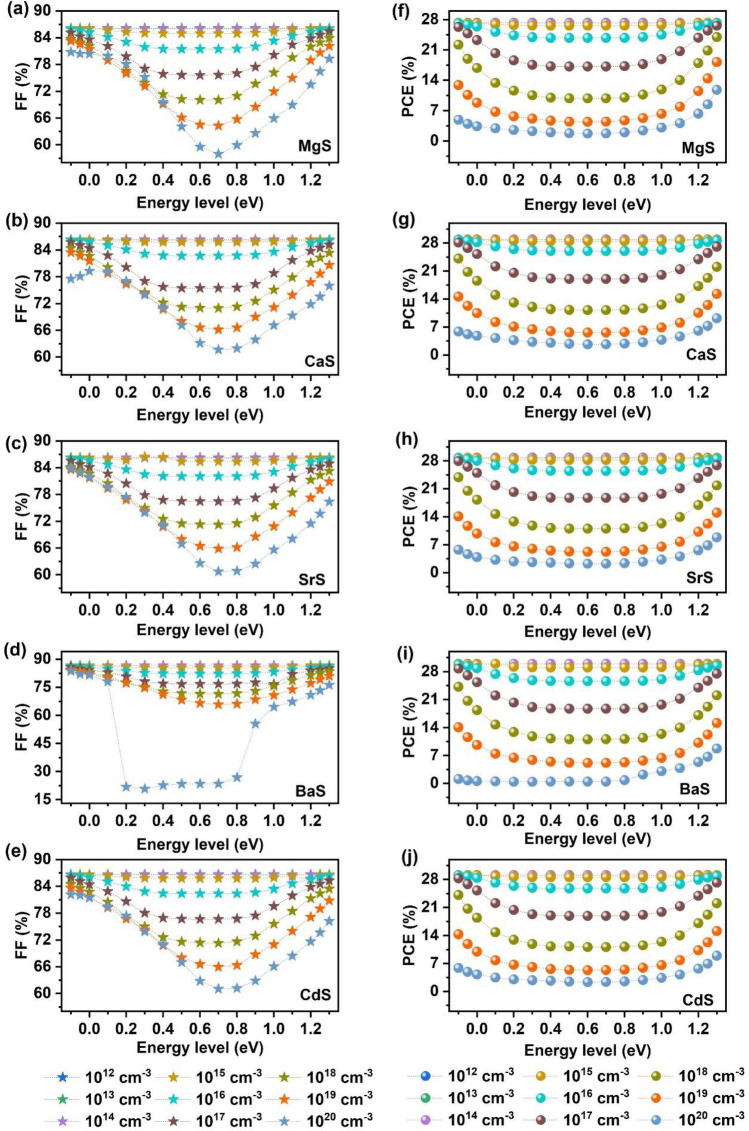
Effect of absorber’s defect energy level and density on (**a–e**) FF and (**f–j**) PCE of novel Ag_2_BaTiSe_4_ solar cells with diverse buffers.

In addition, it is important to study the influence of absorber’s shallow and deep level defects on the performance of solar cells. Hence, we investigated the impact of varying the defect location and density in Ag_2_BaTiSe_4_ absorbers on the photovoltaic parameters of solar cells. The observed variation in V_OC_ and J_SC_ are displayed in Fig. 12 while the changes in FF and PCE are provided in Fig. 13. We varied the defect location from − 0.1 to 1.3 eV, corresponding to the valence band and defect density from 10^12^ to 10^20^ cm^−3^. We found that the photovoltaic parameters were independent of the defect's position up to 10^15^ cm^−3^, after which they declined with increasing defects. Shallow-level defects, located near/inside the valence and conduction band, were observed in the regions between − 0.1 and 0.1 eV and 1.1 to 1.3 eV, while deep-level defects were present in the region between 0.1 and 1.1 eV due to their presence away from the energy bands^103^. V_OC_ and FF were almost unchanged at shallow levels near the valence band when the defect density was larger than 10^15^ cm^−3^. However, they slightly decreased when the defects were near the conduction band and drastically fell at the deep levels in all solar cells. Similarly, J_SC_ nearly retained its maximum value at shallow levels when the defect density was increased from 10^15^ to 10^18^ cm^−3^, while it decreased for deep levels. For defects > 10^18^ cm^−3^, it showed a decline, even at shallow levels, where the decrement was more pronounced at the deep levels. PCE was largely dependent on J_SC_ rather than V_OC_ and FF. Shallow-level defects aided in the formation of proper band alignment at the interface of transport layers, enriching charge carrier transportation, and had little effect on PCE until 10^18^ cm^−3^, beyond which they degraded PCE^45^. On the other hand, deep-level defects caused a dramatic reduction in PCE, with the lowest PCEs of 1.71%, 2.67%, 2.24%, 0.45%, and 2.35% in MgS, CaS, SrS, BaS, and CdS-based solar cells respectively, obtained at ~ 0.6 eV for 10^20^ cm^−3^. This occurs because a large number of photogenerated electrons and holes are trapped due to the presence of deep-level defects in the mid-bandgap regions, leading to high recombination and limiting the separation of charge carriers to the transport layers, degrading the overall performance of solar cells^45,104^. Therefore, to achieve the highest PCE, the deep-level and shallow-level defects in Ag_2_BaTiSe_4_ absorbers must be less than 10^15^ cm^−3^ and 10^18^ cm^−3^, respectively.

### Optimization of MoSe_2_

In I_2_-II-IV-VI_4_ thin film solar cells, the formation of MoSe_2_ is inevitable at the absorber/Mo interface due to the requisite selenization process^105^. MoSe_2_ belongs to the transition metal dichalcogenide semiconductor family which possess excellent structural, physical and optoelectronic properties owing to its 2D structure^106^. It displays both n and p-type characteristics. In practice, it has been found that p-MoSe_2_ is helpful for improving solar cell performance as it forms ohmic contact at the junction between the absorber and Mo. Conversely, n-MoSe_2_ creates a Schottky barrier that enhances back contact recombination^107^. Y. Song et al. have improved the p-type conductivity of MoSe_2_ by doping Nb and have shown that p-MoSe_2_ reduces the barrier for hole collection at the back contact and suppresses interfacial recombination at the absorber/Mo junction^108^. Thus, to understand the role of p-MoSe_2_ properties on the performance of novel Ag_2_BaTiSe_4_ solar cells, its thickness and carrier concentration are varied from 0.050 to 0.2 µm and 10^12^ to 10^20^ cm^−3^ respectively.

#### Effect of MoSe_2_’s thickness and carrier concentration

**Figure 14:**
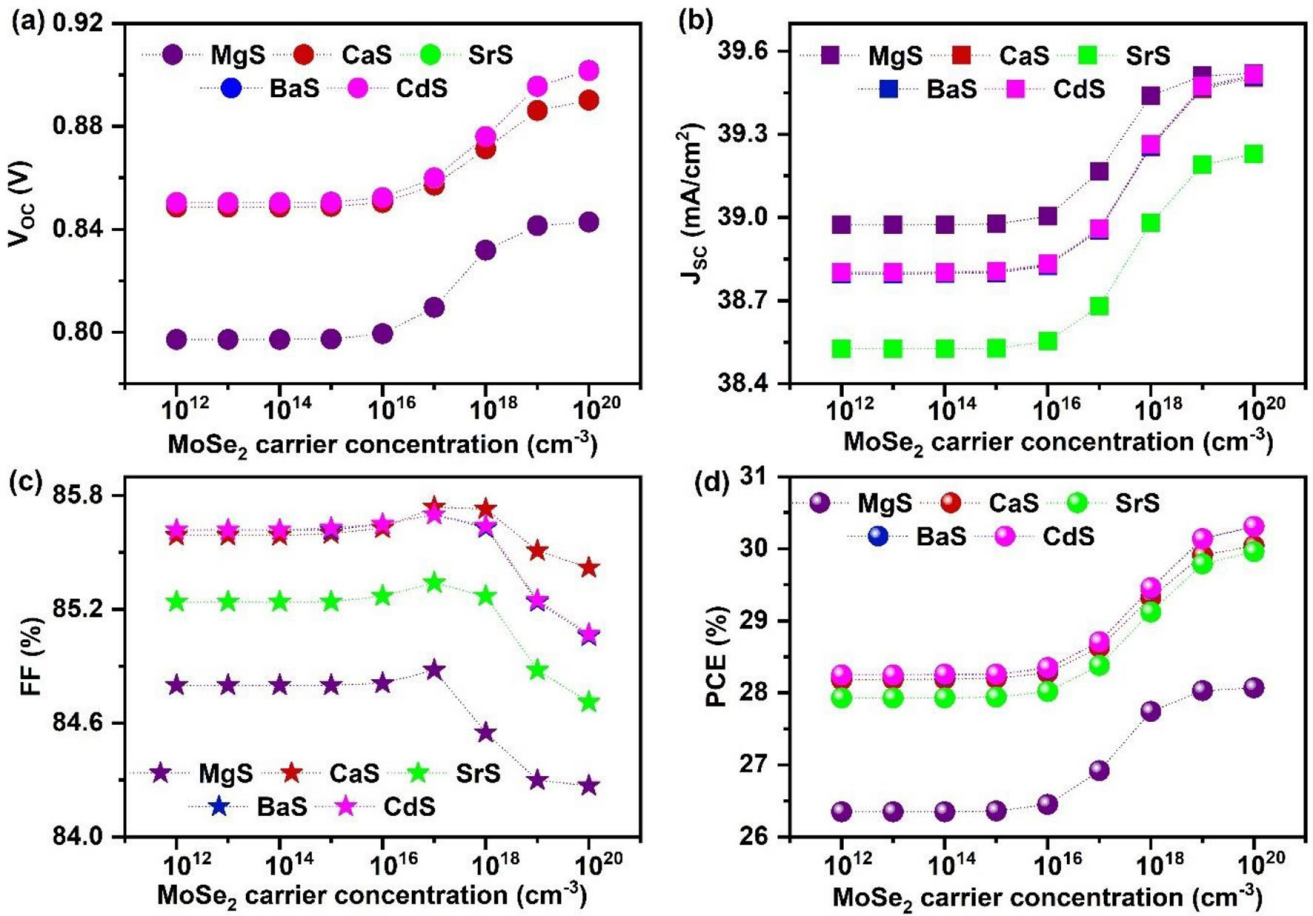
Effect of MoSe_2_’s carrier concentration on (**a**) V_OC_ (**b**) J_SC_ (**c**) FF (**d**) PCE of novel Ag_2_BaTiSe_4_ solar cells with diverse buffers.

**Figure 15:**
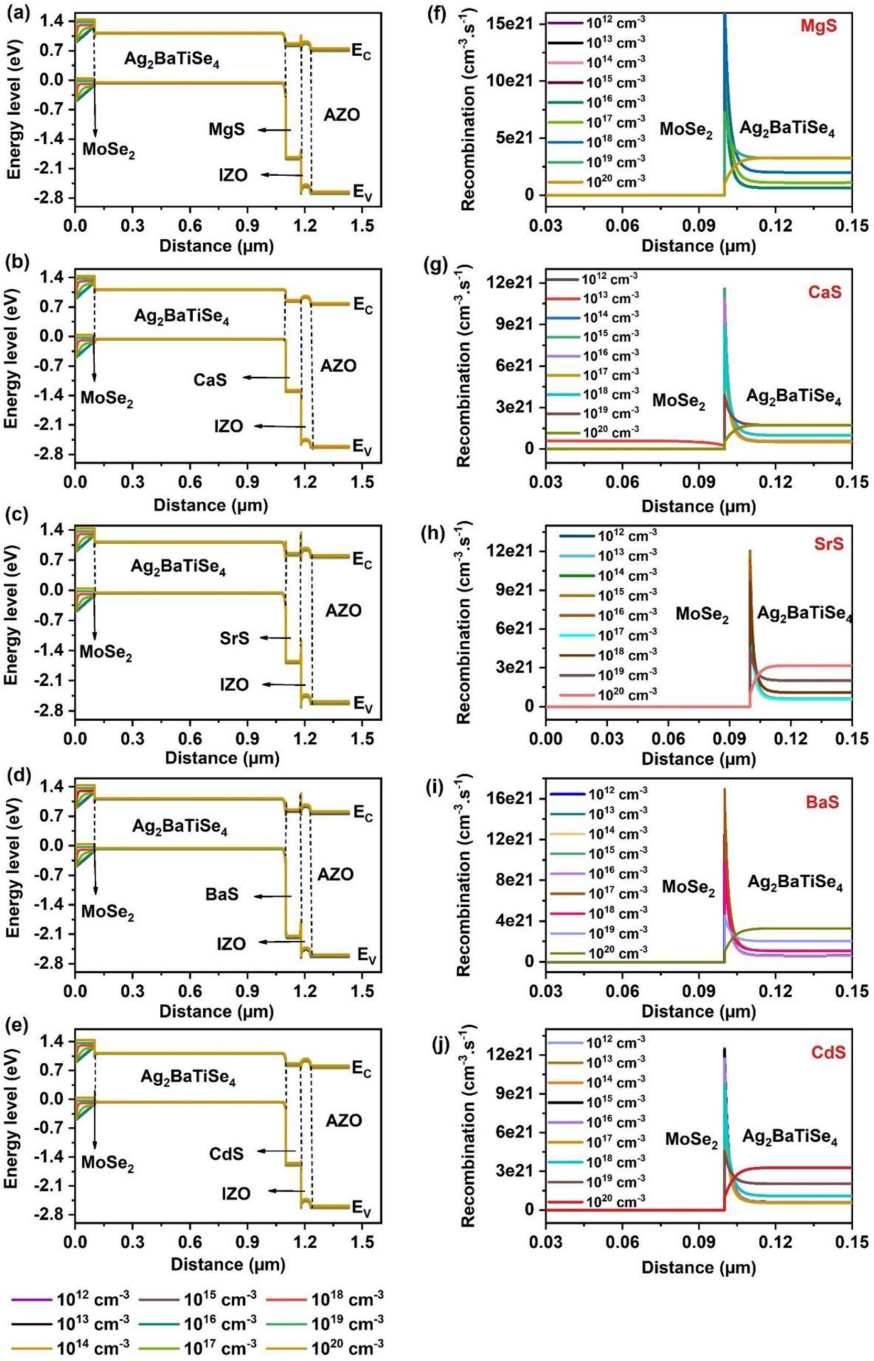
Effect of MoSe_2_’s carrier concentration on (**a–e**) Energy band alignment and (**f–j**) Recombination rate at Ag_2_BaTiSe_4_/MoSe_2_ interface in novel Ag_2_BaTiSe_4_ solar cells with diverse buffers.

Optimizing the thickness of MoSe_2_ is critical for improving the performance of solar cells. When the MoSe_2_ layer is very thin, it increases recombination near the back contact. On the other hand, if the MoSe_2_ layer is too thick, it reduces the thickness of the Mo layer, degrading the electrical contact of the absorber to Mo and increasing the R_S_ in the solar cells^105,109^. Thus, in this study, the MoSe_2_ thickness was varied from 0.050 to 0.2 µm in all solar cells to determine the optimal value (Fig. S5). However, it was found that the variations in the V_OC_, J_SC_, FF, and PCE with respect to MoSe_2_’s thickness were insignificant. According to literature reports, the thickness of MoSe_2_ in I_2_-II-IV-VI_4_ solar cells is typically in the range of a few 0.1 µm to ~ 1 µm at the interface of absorber/Mo^96,105,109,110^. However, high PCEs were reported for thicknesses in the range of ~ 0.1 to 0.2 µm, indicating that the R_S_ and back contact recombination could be minimized in this range^7,52^. Therefore, considering the material cost and overall performance, 0.1 µm is considered the optimal value for further simulations. Thereafter, the influence of MoSe_2_’s carrier concentration on the characteristics of the solar cells is analyzed by differing it from 10^12^ to 10^20^ cm^−3^. Figure 14 demonstrates the changes in solar cell parameters as a function of MoSe_2_’s carrier concentration. The corresponding J–V is given in Fig. S6. It can be observed that all solar cells’ V_OC_, J_SC_, and PCE almost remain constant till 10^16^ cm^−3^ and then improve to higher values. Conversely, FF drops after 10^17^ cm^−3^ in all solar cells. This observance can be elucidated by the changes in the band alignment corresponding to MoSe_2_’s carrier concentration (Fig. 15a–e). No change in energy bands is noticed for the concentrations 10^12^–10^16^ cm^−3^, referring to the constant solar cell performance till the mentioned range. This also reveals that MoSe_2_’s carrier concentration must be greater than 10^16^ cm^−3^ to contribute to the solar cell performance significantly. Interestingly, both E_C_ and E_V_ of MoSe_2_ shift upwards for concentrations > 10^16^ cm^−3^, in consequence of which the barrier for holes is gradually diminished while the electron’s barrier is boosted at the Ag_2_BaTiSe_4_/MoSe_2_ interface. This facilitates the transportation of holes from Ag_2_BaTiSe_4_ to MoSe_2_ while restricting the flow of electrons. As a result, the probability of interface recombination declines, which is also witnessed in Fig. 15f–j, where the recombination rate of charge carriers at the Ag_2_BaTiSe_4_/MoSe_2_ interface as a function of MoSe_2_’s carrier concentration is depicted. The figure shows that as the carrier concentration increases, the recombination rate at the Ag_2_BaTiSe_4_/MoSe_2_ interface drastically falls in all solar cells due to the attainment of appropriate barriers for electrons and holes. This has led to an improvement in V_OC_. In addition, the strong built-in electric field will also be generated at the Ag_2_BaTiSe_4_/MoSe_2_ interface with increasing MoSe_2_’s carrier concentration, which promotes the collection of holes at the back contact without recombination, causing an enhancement in J_SC_^111,112^. However, the R_S_ may increase at higher concentrations, resulting in FF decrement^113^. Nevertheless, the elevation in V_OC_ and J_SC_ has boosted the PCE from 26.35 to 28.07%, 28.18 to 30.04%, 27.93 to 29.96%, 28.25 to 30.31%, and 28.26 to 30.31% in MgS, CaS, SrS, BaS and CdS-based solar cells respectively. Thus, MoSe_2_’s carrier concentration of 10^20^ cm^−3^ is the optimum value for achieving high PCE in all solar cells.

### Effect of defects at Ag_2_BaTiSe_4_/buffer and MoSe_2_/Ag_2_BaTiSe_4_ interface

**Figure 16:**
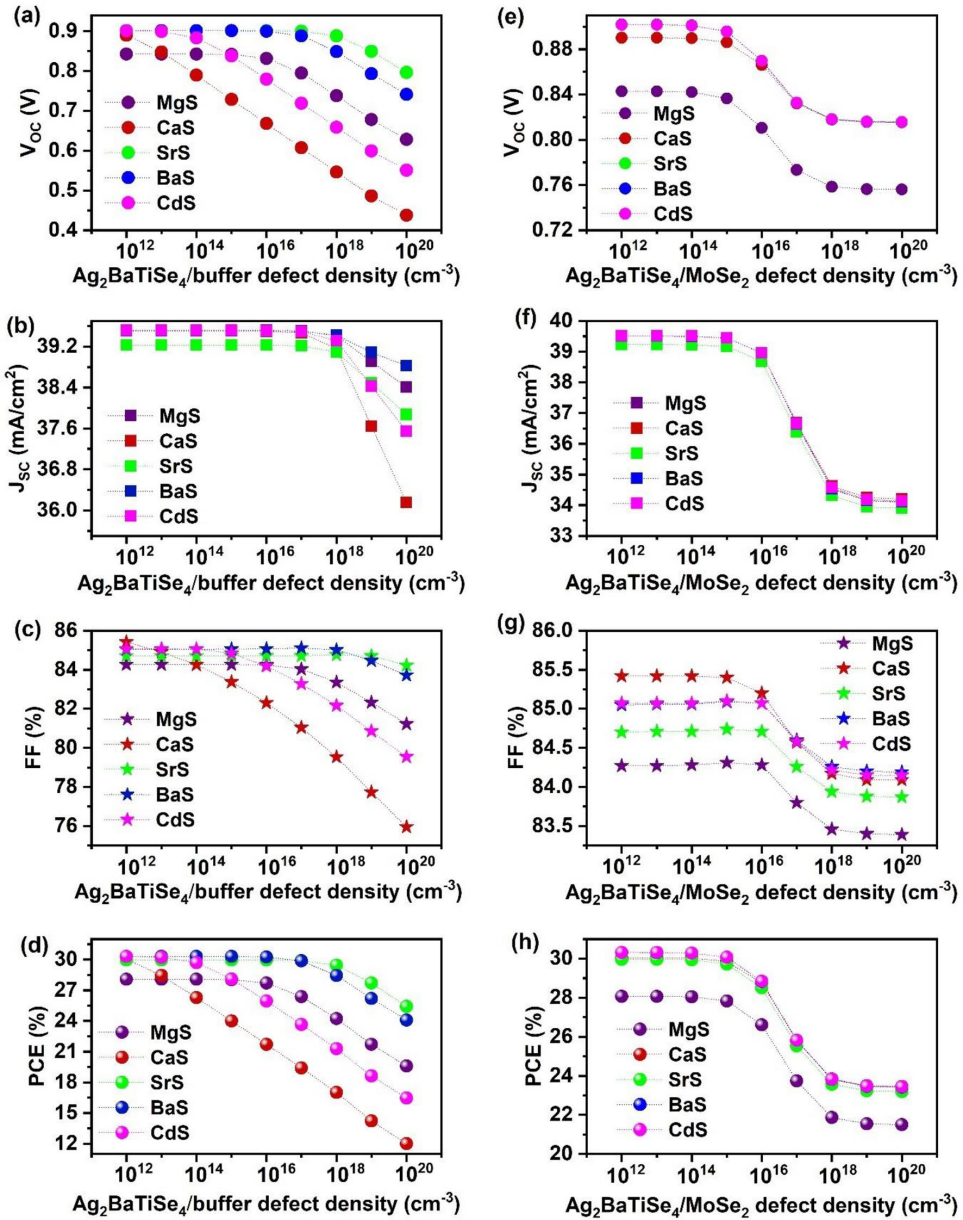
Effect of defect density at Ag_2_BaTiSe_4_/buffer and Ag_2_BaTiSe_4_/MoSe_2_ interface on the photovoltaic parameters of novel Ag_2_BaTiSe_4_ solar cells with diverse buffers.

Interface defects are inevitable in solar cells, which are formed during fabrication due to structural imperfections. These defects boost the interface recombination of charge carriers that are detrimental to solar cell performance^67^. Thus, it is crucial to analyze their influence on these solar cells and obtain the optimum value to fabricate them practically. So far, all the simulations were performed with the neutral interface defect density of 10^12^ cm^−3^ at Ag_2_BaTiSe_4_/buffer and Ag_2_BaTiSe_4_/MoSe_2_ interface where the defect levels are fixed at 0.6 eV above E_V_ in all solar cells. Herein, the impact of interface defect density on the photovoltaic parameters is estimated by varying it from 10^12^ to 10^20^ cm^−3^ at both interfaces. The respective variations in J–V characteristics are depicted in Fig. S7. Figure 16a–d demonstrates the outcomes of all solar cells regarding the Ag_2_BaTiSe_4_/buffer interface defects. V_OC_ and FF are approximately the same till 10^15^ cm^−3^, 10^17^ cm^−3^, 10^16^ cm^−3^, and 10^14^ cm^−3^ for MgS, SrS, BaS, and CdS-based solar cells, respectively, and decline afterward. In the case of CaS-based solar cells, they drastically reduce beyond 10^12^ cm^−3^. In contrast, J_SC_ remains stable till 10^18^ cm^−3^ in all solar cells and then decreases. However, the reduction in J_SC_ is less significant compared to V_OC_ and FF. Moreover, the PCE of all solar cells follows a similar trend as V_OC_ and FF, indicating that the overall performance of all solar cells is determined mainly by the changes in V_OC_ and FF while less influenced by J_SC_ at Ag_2_BaTiSe_4_/buffer interface. The level of degradation in PCE is observed to be 8.48%, 18.03%, 4.55%, 6.22%, and 13.86% in MgS, CaS, SrS, BaS, and CdS-based solar cells, respectively, for the defect density range 10^12^–10^20^ cm^−3^. This clearly reveals that CaS and CdS-based solar cells are more sensitive to the Ag_2_BaTiSe_4_/buffer interface defects than the other solar cells. In contrast, SrS-based solar cell is comparatively stable with the defects. The overall degradation in solar cell performance with the interface defects occurs due to the increasing trap-assisted recombination of photogenerated electrons at the buffer/Ag_2_BaTiSe_4_ interface, which restricts their flow towards the front contact^114^. Thus, the optimum defect density of 10^15^ cm^−3^, 10^12^ cm^−3^, 10^17^ cm^−3^, 10^16^ cm^−3^, and 10^14^ cm^−3^ are selected for MgS, CaS, SrS, BaS, and CdS-based solar cells, respectively at Ag_2_BaTiSe_4_/buffer interface to attain maximum solar cell performance. These values are higher than the defect densities reported in the experiments at the CZTS/buffer interface, revealing the superiority of novel Ag_2_BaTiSe_4_ solar cells over their predecessors^115,116^. Similarly, the defect density at the Ag_2_BaTiSe_4_/MoSe_2_ interface is shifted from 10^12^ to 10^20^ cm^−3^ (Fig. 16e–h). Here, all the solar cell parameters start to reduce for defect density above 10^15^ cm^-3^ and begin to saturate at 10^18^ cm^−3^. It can be noticed that J_SC_ is adversely affected than V_OC_ and FF, i.e., it decreases from ~ 39 to ~ 34 mA cm^−2^ when defect density is raised from 10^12^ to 10^18^ cm^−3^. This has led to a decline in PCE by ~ 6.5% in all solar cells. The drop in the solar cell performance may happen because holes travelling from Ag_2_BaTiSe_4_ to MoSe_2_ have a high chance of being trapped or recombined when the defects at the Ag_2_BaTiSe_4_/MoSe_2_ interface increases^114^. The results show that defect density < 10^15^ cm^−3^ is required at the Ag_2_BaTiSe_4_/MoSe_2_ interface for exceptional solar cell performance. Overall, the investigation of interface defects suggests that it has a massive impact on solar cell performance. These defects are generally produced due to the structural defects between different layers and metal cation diffusion through the absorber during fabrication^67^. Thus, effective techniques must be employed for the deposition of layers, and methods such as etching, post-heat treatment, and inserting a passivation layer can be used to minimize the interface defects in solar cells^117–119^.

**Figure 17:**
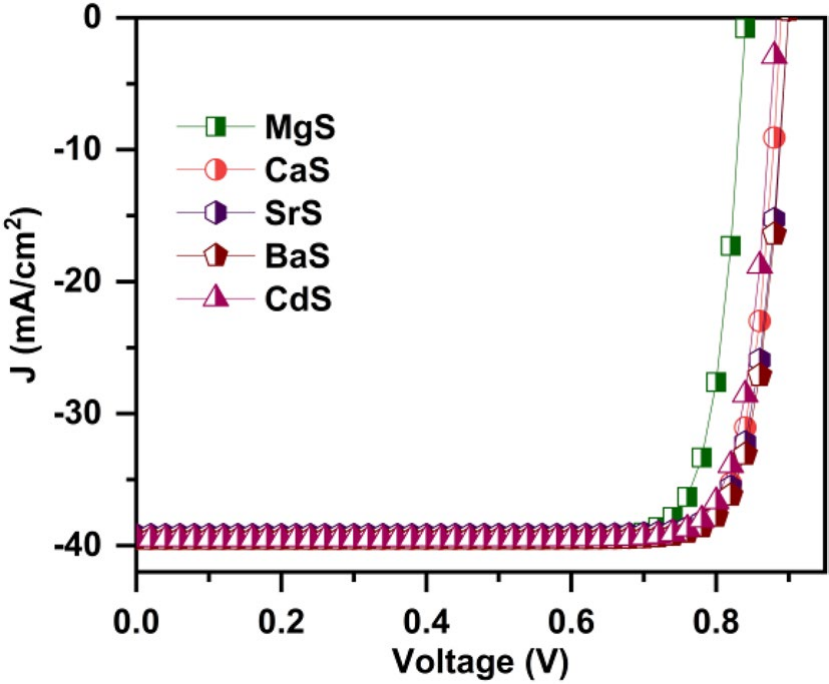
J–V graph of novel Ag_2_BaTiSe_4_ solar cells with diverse buffers after optimization.

Table 5 displays the final solar cell performance of the novel Ag_2_BaTiSe_4_ solar cells with diverse buffers after optimization, and the corresponding J-V is displayed in Fig. 17. As discussed in the introduction, the main problem that limits the PCE of I_2_-II-IV-VI_4_ solar cells is their large V_OC_ deficit. In addition, CdS is used as a buffer in most solar cells containing the toxic element Cd, creating severe problems when dumped into the environment. Therefore, developing efficient absorbers and eco-friendly buffers is always highly interesting to the photovoltaic community. In this regard, for the first time, we have reported Ag_2_BaTiSe_4_ of group I_2_-II-IV-VI_4_ as a potential absorber and a new class of alkaline earth metal chalcogenides, namely MgS, CaS, SrS, and BaS as alternative buffers using SCAPS-1D. Herein, we have accomplished high PCEs of 28.00%, 30.02%, 29.87%, 30.23%, and 29.68% for MgS, CaS, SrS, BaS, and CdS-based Ag_2_BaTiSe_4_ solar cells, respectively. Surprisingly, the PCEs achieved in Ag_2_BaTiSe_4_ solar cells with alkaline earth metal chalcogenides buffers are comparable with CdS, proving their potential and suitability to be applied as alternative, non-toxic buffers in thin-film solar cells. Moreover, the V_OC_ loss in these solar cells is less, specifically 0.3 V, 0.29 V, 0.34 V, 0.28 V, and 0.29 V for MgS, CaS, SrS, BaS, and CdS-based Ag_2_BaTiSe_4_ solar cells respectively, displaying the superior properties of Ag_2_BaTiSe_4_ as an alternative absorber. The low V_OC_ deficit in alkaline earth metal chalcogenides buffer-based Ag_2_BaTiSe_4_ solar cells is highly possible in experiments due to the proper band alignment at absorber/buffer interface and low antisite defects in Ag_2_BaTiSe_4_ absorber because of the large atomic size difference between the constituent elements. Thus, this work would kindle the photovoltaic community’s interest in fabricating novel efficient Ag_2_BaTiSe_4_ solar cells with new alkaline earth metal chalcogenides buffers and achieve high PCE. In addition, based on our research outcomes, we propose that these new alkaline earth metal chalcogenides buffers have immense potential to be utilized in other conventional solar cells such as CdTe, CIGSSe, perovskites, etc.

**Table 5:** Solar cell parameters of novel Ag_2_BaTiSe_4_ solar cells with diverse buffers after optimization.

Solar cell structure	V_OC_ (V)	J_SC_ (mA cm^−2^)	FF (%)	PCE (%)
AZO/IZO/MgS/Ag_2_BaTiSe_4_/MoSe_2_/Mo	0.840	39.51	84.28	28.00
AZO/IZO/CaS/Ag_2_BaTiSe_4_/MoSe_2_/Mo	0.889	39.49	85.42	30.02
AZO/IZO/SrS/Ag_2_BaTiSe_4_/MoSe_2_/Mo	0.899	39.20	84.72	29.87
AZO/IZO/BaS/Ag_2_BaTiSe_4_/MoSe_2_/Mo	0.899	39.50	85.07	30.23
AZO/IZO/CdS/Ag_2_BaTiSe_4_/MoSe_2_/Mo	0.883	39.50	85.06	29.68

Overall, after the optimization of buffer, Ag_2_BaTiSe_4_, MoSe_2_, and interface properties, the PCE incredibly improved from 18.72%, 11.65%, 15.93%, 15.47% and 14.99% to 28.00%, 30.02%, 29.87%, 30.23% and 29.68% for MgS, CaS, SrS, BaS and CdS-based Ag_2_BaTiSe_4_ solar cells respectively. Further, the final solar cells where the high PCEs have been demonstrated are selected for the upcoming studies to investigate the impact of R_S_, R_Sh_, and working temperature on their performance.

### Effect of series and shunt resistances

**Figure 18:**
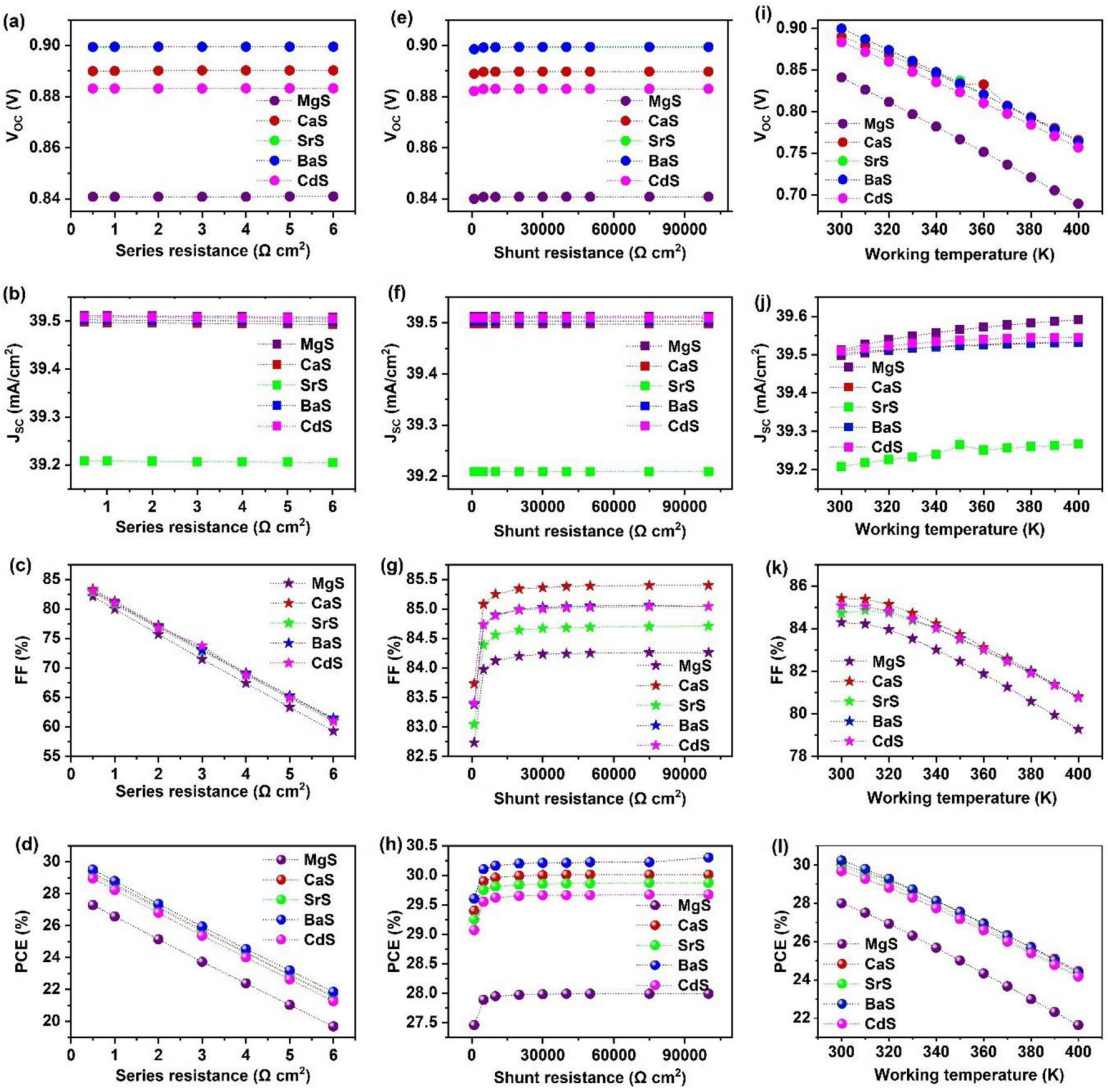
Effect of series resistance, shunt resistance, and working temperature on the performance of novel Ag_2_BaTiSe_4_ solar cells with diverse buffers.

R_S_ and R_Sh_ display significant impact on the performance of solar cells. R_S_ is the sum of resistance at the front and back contacts and between various layers of solar cells. On the other hand, R_Sh_ originates from the reverse saturation current in solar cells that is produced by the manufacturing defects^120^. Here, the influence of R_S_ and R_Sh_ on the solar cell parameters is investigated using SCAPS-1D. Figure 18a–d displays V_OC_, J_SC_, FF, and PCE as a function of R_S_, where it is varied from 0.5 to 6 Ω cm^2^ for all solar cells. The respective variations in J–V characteristics are depicted in Fig. S8. It can be observed that V_OC_ and J_SC_ remain unaffected throughout the R_S_ range. Whereas, FF drastically decreases from 82.12 to 59.27%, 83.34 to 61.27%, 82.72 to 61.35%, 83.05 to 61.49%, and 82.99 to 60.96% in MgS, CaS, SrS, BaS and CdS-based solar cells respectively. The massive reduction in FF is attributed to the colossal power loss in the solar cells with increasing R_S_, which adversely affects their performance^121^. Thus, when R_S_ is improved from 0.5 to 6 Ω cm^2^, PCE dramatically declined by ~ 7.5% in all the solar cells. Similarly, R_Sh_ is tuned from 1000 to 100,000 Ω cm^2^ in all solar cells, as shown in Fig. 18e–h. The respective changes in J–V are depicted in Fig. S8. In this case, V_OC_ and J_SC_ are almost the same for all R_Sh_ values. On the contrary, the FF and PCE values enhanced with an increment in the R_Sh_ till 2000 Ω cm^2^ and saturate on further increase in R_Sh_. Nevertheless, the rise in PCE is about 0.53%, 0.61%, 0.62%, 0.7%, and 0.6% in MgS, CaS, SrS, BaS, and CdS-based solar cells, respectively. This exhibits that the effect of R_Sh_ on the performance of these solar cells is negligible compared to R_S_. Therefore, a low R_S_ of 0.5 Ω cm^2^ is highly recommended for the efficient functioning of these novel Ag_2_BaTiSe_4_ solar cells.

### Effect of working temperature

Long-term stability in the environment is an essential requirement for the application of solar cells^122^. Thus, the deterioration process of solar cells under ambient air conditions must be investigated. In the present study, the working temperature is varied from 300 to 400 K for analyzing its influence on all solar cells. Figure 18i–l demonstrates the response of solar cells concerning working temperature. The corresponding variations in J–V graphs are depicted in Fig. S8. It can be seen that V_OC_, FF, and PCE diminish with increasing temperature while J_SC_ slightly increases in all solar cells. The temperature rise reduces the bandgap of the absorber, which enhances the charge carrier generation in solar cells, leading to enhancement in J_SC_^122^. However, the thermally generated electrons begin to vibrate at high temperatures, become unstable, and recombine with the holes before being collected at the contacts, reducing V_OC_. Moreover, the increasing temperature affects the transport efficiency of charge carriers, such as carrier concentration and mobility of charge carriers, thereby decreasing the FF of all solar cells^123^. Overall, the combined decrement in V_OC_ and FF has led to a lowering of PCE from 28.0% to 21.64%, 30.02 to 24.46%, 29.87 to 24.23%, 30.23 to 24.39%, 29.68 to 24.17% for MgS, CaS, SrS, BaS, and CdS-based solar cells respectively.

## Conclusion

In the present work, a comprehensive numerical study on the performance of novel Ag_2_BaTiSe_4_ solar cells with new alkaline earth metal chalcogenides as alternative buffers to CdS was performed using SCAPS-1D. Upon optimizing buffer properties, maximum PCE of 18.84%, 17.17%, 20.65%, 20.87%, and 18.66% for MgS, CaS, SrS, BaS, and CdS-based respective solar cells were obtained, which mainly originated from the variation in N_D buffer_. The energy bands shifted downwards for N_D buffer_ > N_A absorber_, diminishing the barrier height at the absorber/buffer interface, eventually suppressing the recombination rate and enhancing the V_b_ and W of solar cells. Thereafter, at the optimized absorber’s electron affinity (4.4 eV) and thickness (1 µm), PCE improved by 5.33% for CaS-based solar cells and ~ 2% for other solar cells due to the increment in R_rec_ and light absorption of solar cells, as evidenced by Nyquist plots and QE measurements. Notably, the absorber’s carrier concentration of 10^18^ cm^−3^ drastically escalated the performance of all solar cells, while its defect density > 10^15^ cm^−3^ declined the PCE owing to the dramatic decrease in the τ and L of charge carriers. Further, on tuning MoSe_2_ parameters and interfacial properties, the best PCE of 28%, 30.02%, 29.87%, 30.23%, and 29.68% was achieved with minimal V_OC_ loss of ~ 0.3 V for MgS, CaS, SrS, BaS, and CdS-based solar cells respectively. Therefore, this work could open constructive research avenues for the photovoltaic community to fabricate highly efficient thin-film solar cells using novel Ag_2_BaTiSe_4_ as an absorber and new alkaline earth metal chalcogenides as alternative, non-toxic buffers.

### Supplementary Information

Supplementary Figures.

## Data Availability

The data that support the findings of this study are provided in the supplementary material of this article, and further required data are available from the corresponding author upon reasonable request.
