# Supplementary material for: Highly efficient emerging Ag2BaTiSe4 solar cells using a new class of alkaline earth metal-based chalcogenide buffers alternative to CdS
Source: Sci Rep. 2024 Jan 17;14:1473. doi: 10.1038/s41598-024-51711-6 (PMC10794422; doi:10.1038/s41598-024-51711-6)
Supplement: Supplementary file 1 — Supplementary Figures. [file 41598_2024_51711_MOESM1_ESM.docx]

**Supplementary information**

**Highly efficient emerging Ag_2_BaTiSe_4_ solar cells using a new class of alkaline earth metal-based chalcogenide buffers alternative to CdS**

Kaviya Tracy Arockiya Dass^1^, M. Khalid Hossain^2^ & Latha Marasamy^1^*

^1^Facultad de Química, Materiales-Energía, Universidad Autónoma de Querétaro, Santiago de Querétaro, Querétaro, C.P.76010, México.

^2^Institute of Electronics, Atomic Energy Research Establishment, Bangladesh Atomic Energy Commission, Dhaka 1349, Bangladesh.

*Corresponding author Email-id: [latha.marasamy@uaq.mx](mailto:latha.marasamy@uaq.mx)


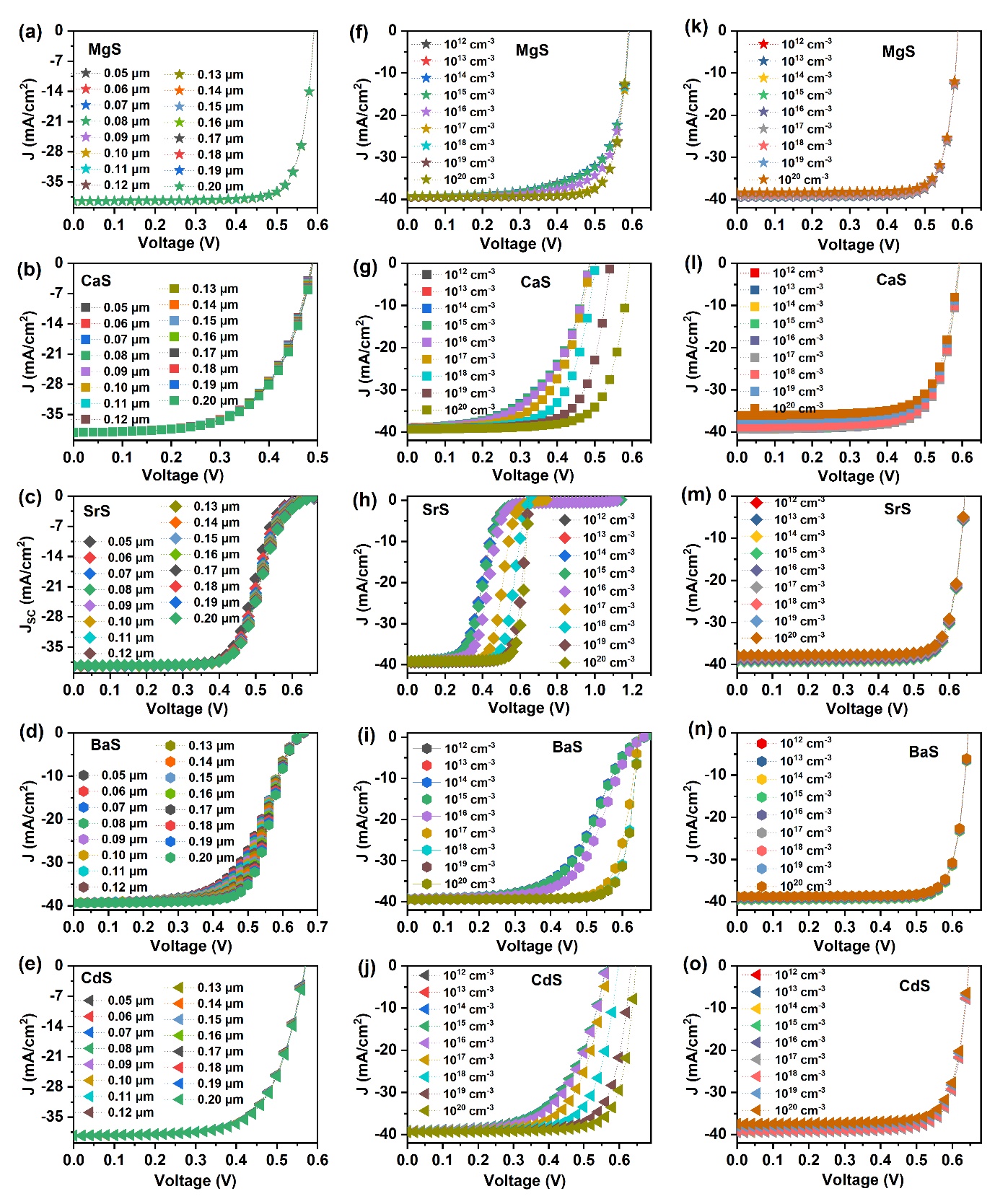


**Figure S1**. J-V graphs of novel Ag_2_BaTiSe_4_ solar cells with diverse buffers corresponding to the variation in buffer’s (**a-e**) thickness, (**f-j**) carrier concentration, and (**k-o**) defect density.


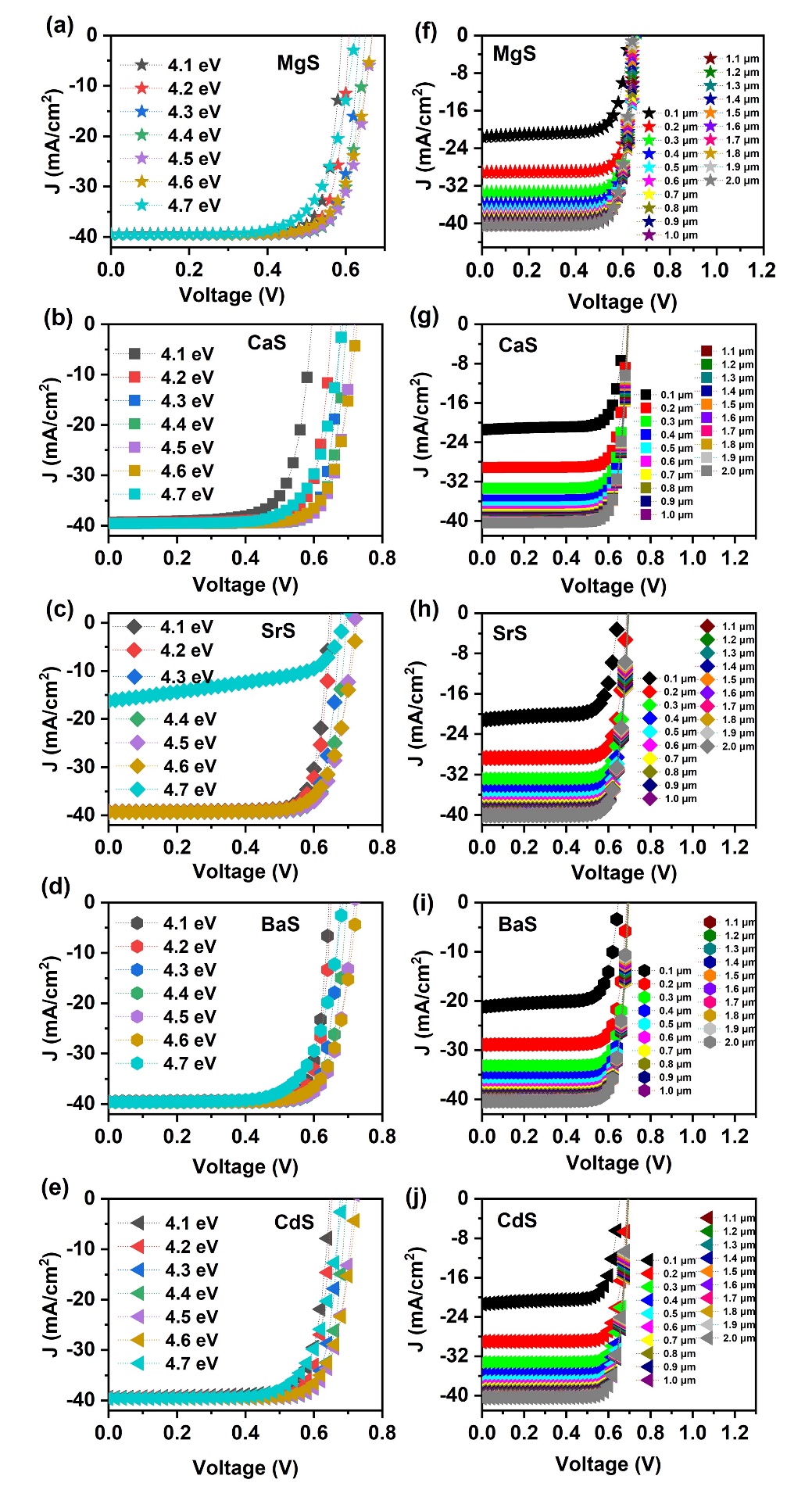


**Figure S2**. J-V graphs of novel Ag_2_BaTiSe_4_ solar cells with diverse buffers corresponding to the variation in absorber’s (**a-e**) electron affinity (**f-j**) thickness.


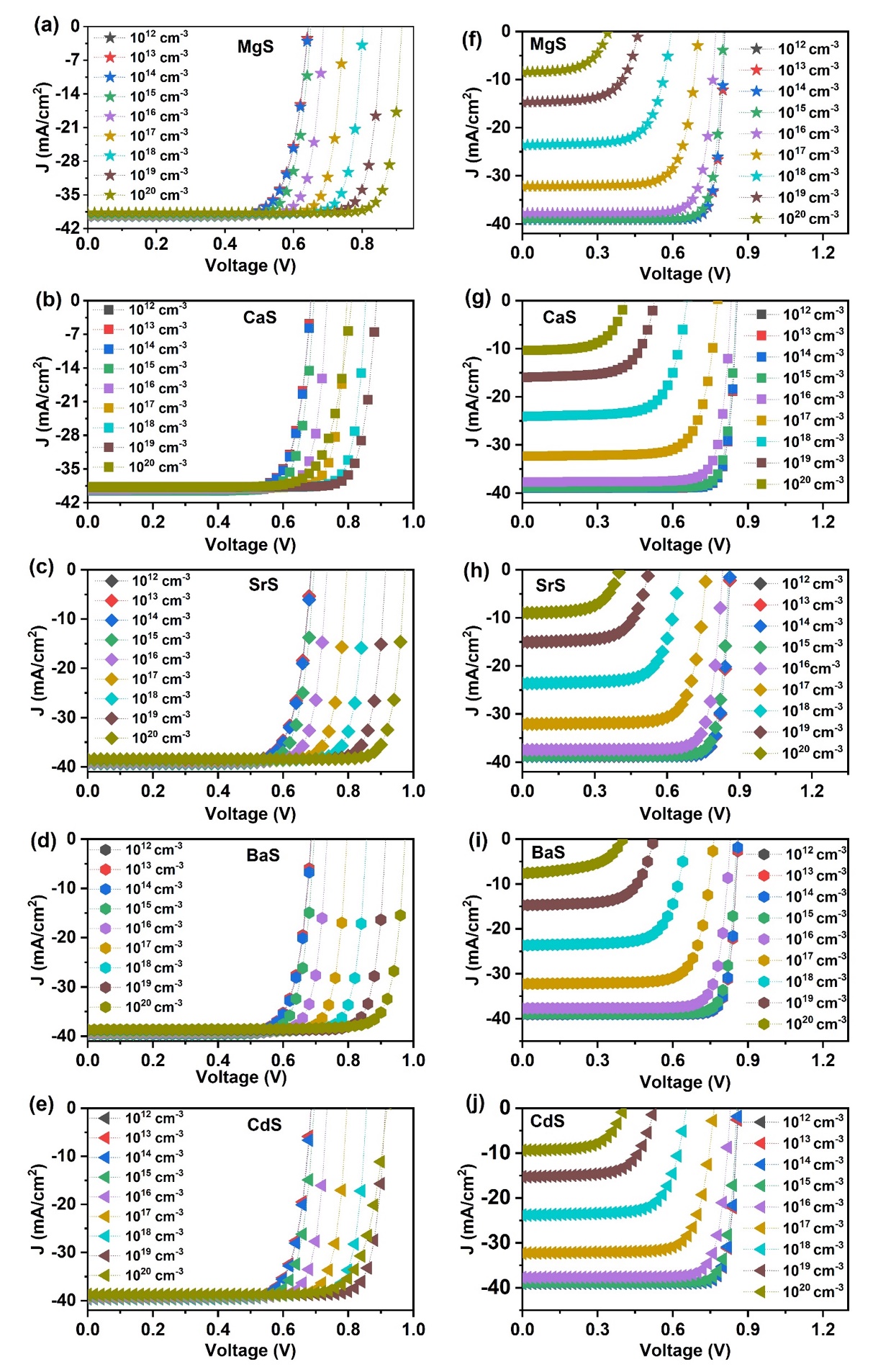


**Figure S3**. J-V graphs of novel Ag_2_BaTiSe_4_ solar cells corresponding to the variation in absorber’s (**a-e**) carrier concentration (**f-j**) Defect density.


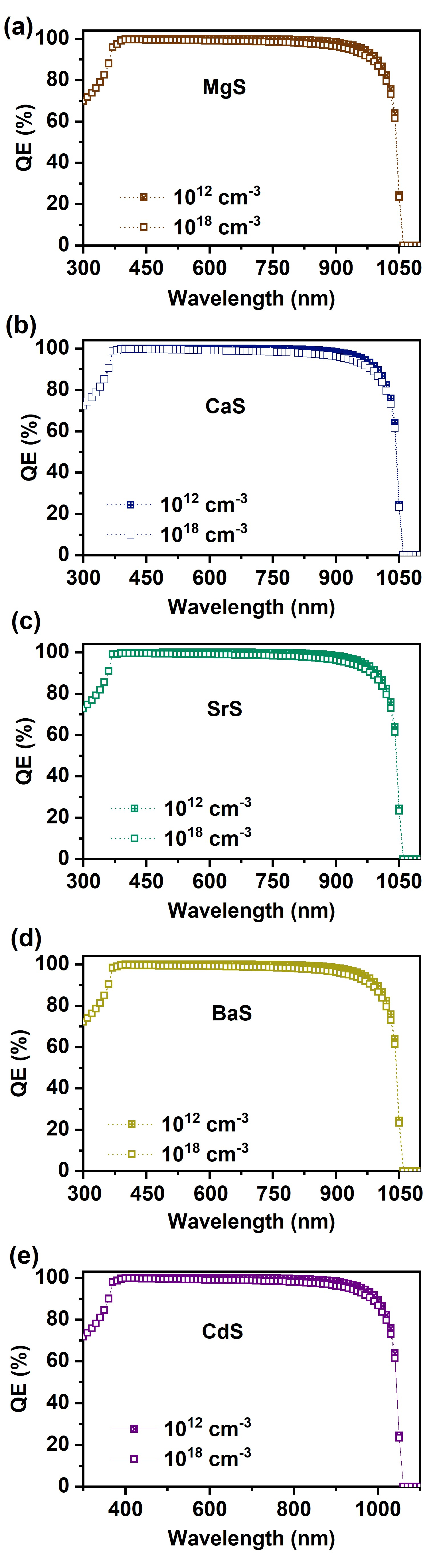


**Figure S4**. Simplified image of changes in QE (magnified image) at absorber’s carrier concentration of 10^12^ and 10^18^ cm^-3^ in novel Ag_2_BaTiSe_4_ solar cells with diverse buffers.


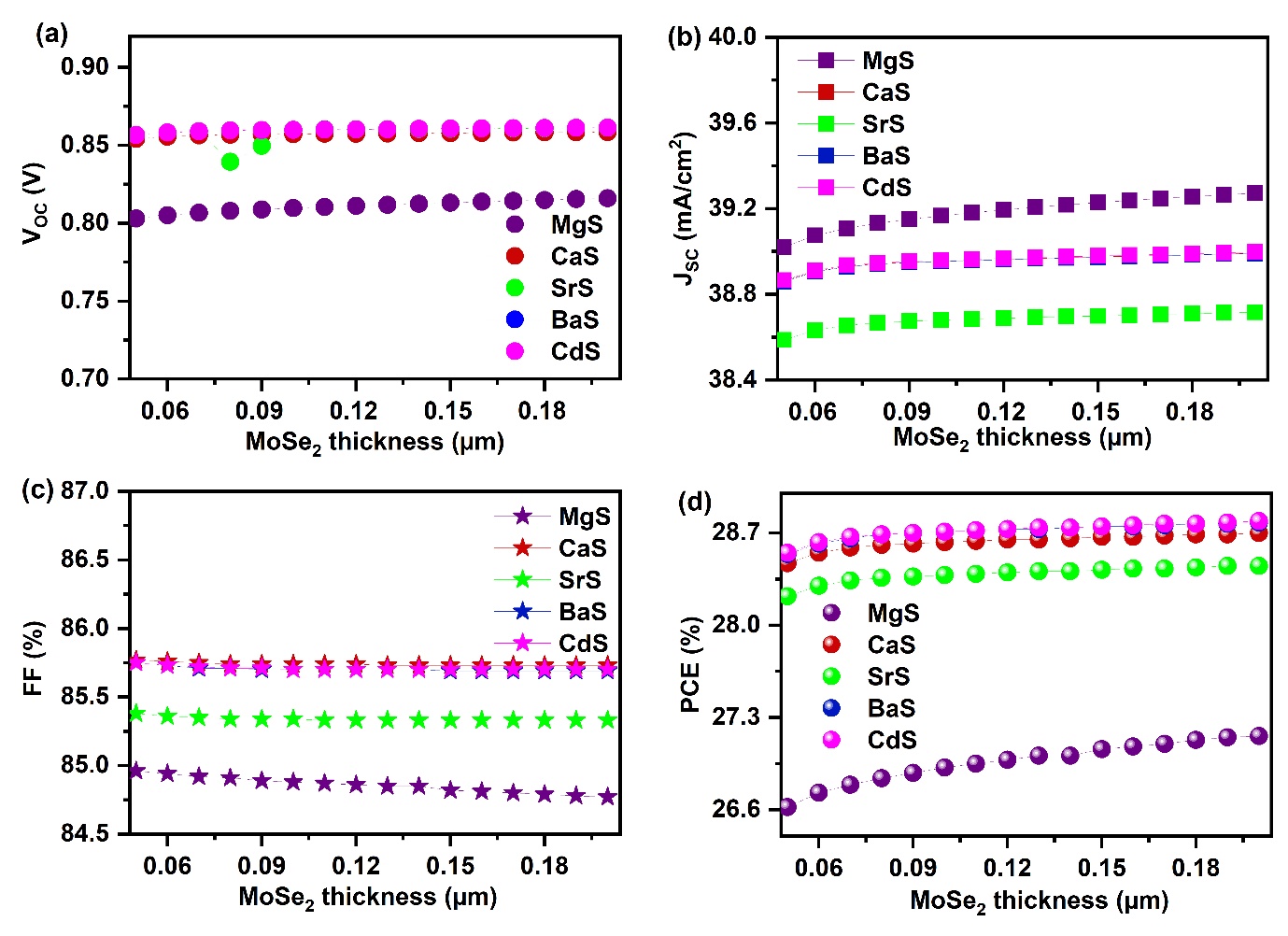


**Figure S5**. Effect of MoSe_2_’s thickness on (**a**) V_OC_ (**b**) J_SC_ (**c**) FF (**d**) PCE of novel Ag_2_BaTiSe_4_ based solar cells.


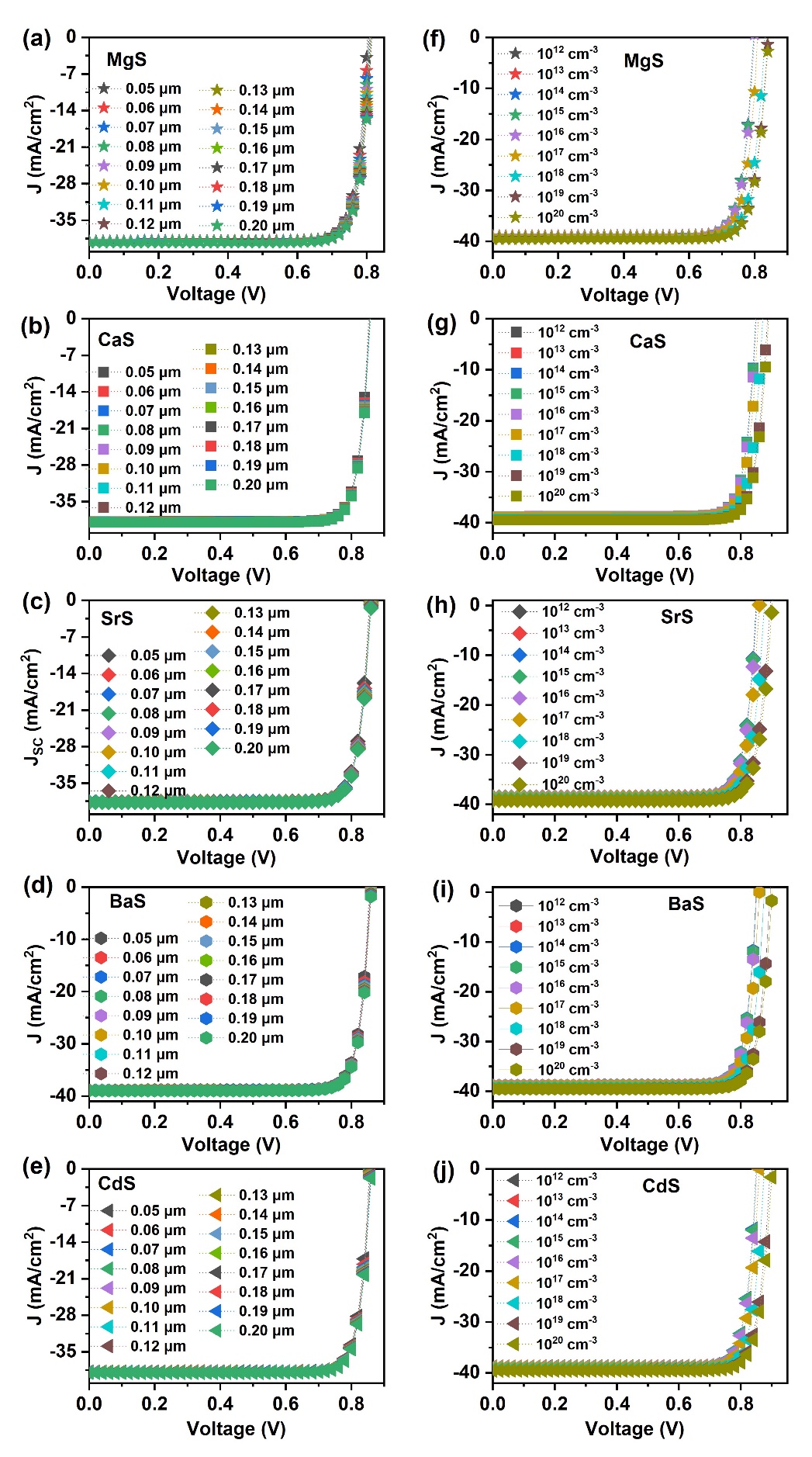


**Figure S6**. J-V graphs of novel Ag_2_BaTiSe_4_ solar cells corresponding to the variation in MoSe_2_’s (**a-e**) thickness (**f-j**) carrier concentration.


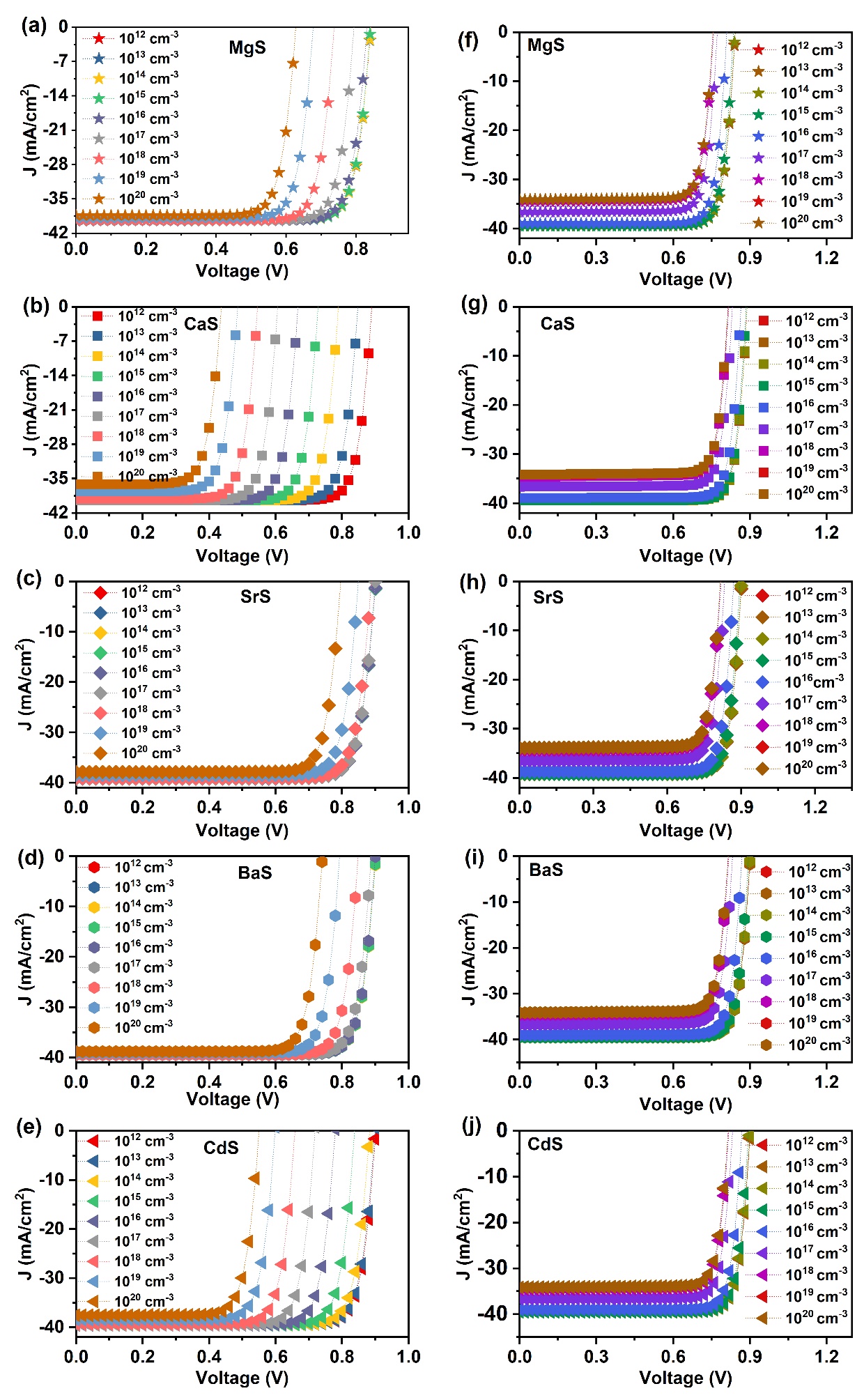


**Figure S7**. J-V graphs of novel Ag_2_BaTiSe_4_ solar cells corresponding to the variation in defect density at (**a-e**) Ag_2_BaTiSe_4_/buffer interface (**f-j**) MoSe_2_/Ag_2_BaTiSe_4_ interface.


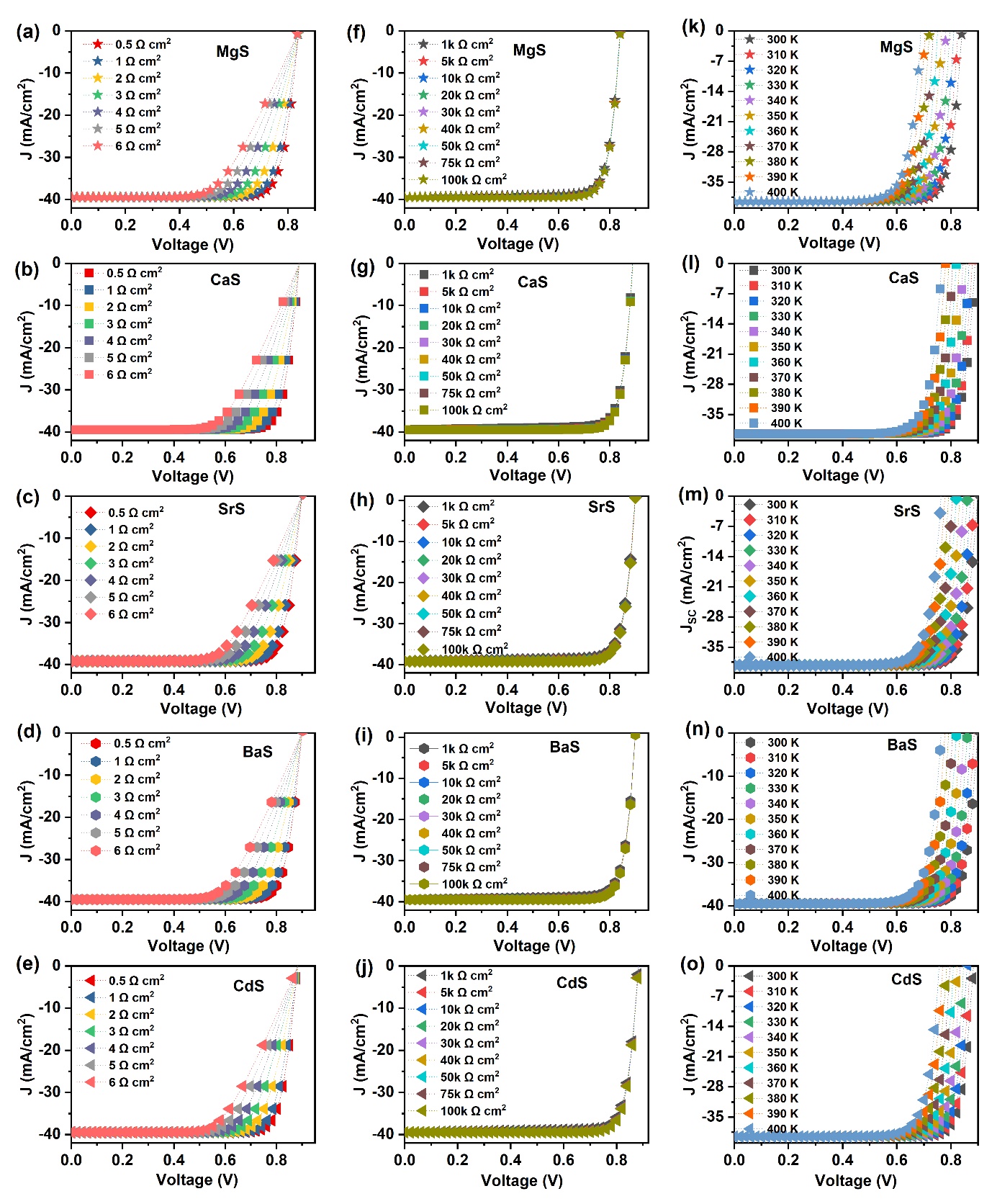


**Figure S8**. J-V graphs of novel Ag_2_BaTiSe_4_ solar cells corresponding to (**a-e**) Series resistance (**f-j**) Shunt resistance (**k-o**) Working temperature.
